# Effectiveness of training general practitioners to improve the implementation of brief stop-smoking advice in German primary care: study protocol of a pragmatic, 2-arm cluster randomised controlled trial (the ABCII trial)

**DOI:** 10.1186/s12875-019-0986-8

**Published:** 2019-07-27

**Authors:** Sabrina Kastaun, Verena Leve, Jaqueline Hildebrandt, Christian Funke, Stephanie Becker, Diana Lubisch, Wolfgang Viechtbauer, Olaf Reddemann, Linn Hempel, Hayden McRobbie, Tobias Raupach, Robert West, Daniel Kotz

**Affiliations:** 1 Institute of General Practice, Addiction Research and Clinical Epidemiology Unit, Medical Faculty of the Heinrich-Heine-University Düsseldorf, Werdener Str. 4, 40227 Düsseldorf, Germany; 2 Department of Psychiatry and Neuropsychology, Maastricht University, Maastricht, Netherlands; 3 Clinical Institute of Psychosomatic Medicine and Psychotherapy, Medical Faculty of the Heinrich-Heine-University Düsseldorf, Düsseldorf, Germany; 4 Wolfson Institute of Preventive Medicine, Queen Mary University of London, London, UK; 5 The Dragon Institute for Innovation, Auckland, New Zealand; 6 Department of Cardiology and Pneumology, University Medical Centre Göttingen, Göttingen, Germany; 7 Research Department of Behavioural Science and Health, Institute of Epidemiology and Health Care, University College London, London, UK; 8 Department of Family Medicine, CAPHRI School for Public Health and Primary Care, Maastricht University, Maastricht, The Netherlands

**Keywords:** Tobacco addiction, Primary care, General practitioner, Brief smoking cessation advice, National practice guideline

## Abstract

**Background:**

The German clinical guideline on tobacco addiction recommends that general practitioners (GPs) provide brief stop-smoking advice to their patients according to the “5A” or the much briefer “ABC” method, but its implementation is insufficient. A lack of training is one barrier for GPs to provide such advice. Moreover, the respective effectiveness of a 5A or ABC training regarding subsequent delivery of stop-smoking advice has not been investigated. We developed a training for GPs according to both methods, and conducted a pilot study with process evaluation to optimize the trainings according to the needs of GPs. This study aims at evaluating the effectiveness of both trainings.

**Methods:**

A pragmatic 2-arm cluster randomised controlled trial with a pre-post data collection will be conducted in 48 GP practices in North Rhine-Westphalia (Germany). GPs will be randomised to receive a 3.5-h-training in delivering either 5A or ABC, including peer coaching and intensive role plays with professional actors. The patient-reported primary outcome (receipt of GP advice to quit: yes/no) and secondary outcomes (recommendation rates of smoking cessation treatments, group comparison (5A versus ABC): receipt of GP advice to quit) will be collected in smoking patients routinely consulting their GP within 4 weeks prior, and 4 weeks following the training. Additional secondary outcomes will be collected at 4, 12 and 26 weeks following the consultation: use of cessation treatments during the last quit attempt (if so) since the GP consultation, and point-prevalence abstinence rates. The primary data analysis will be conducted using a mixed-effects logistic regression model with random effects for the cluster variable.

**Discussion:**

If the training increases the rates of delivery of stop-smoking advice, it would offer a low-threshold strategy for the guideline implementation in German primary care. Should one method prove superior, a more specific guideline recommendation can be proposed.

**Trial registration:**

German Clinical Trials Register (DRKS00012786); registered on 22th August 2017, prior to the first patient in.

**Electronic supplementary material:**

The online version of this article (10.1186/s12875-019-0986-8) contains supplementary material, which is available to authorized users.

## Background

In Germany, over 120,000 people die from smoking each year [1]. About 14% of the country’s mortality is attributable to smoking [1], with approximately one third of tobacco-related deaths occurring during working age, resulting in a huge individual and societal burden [2]. Compared with other European high income countries such as the Netherlands (19%), England (17%), or Sweden (7%) [3], the smoking prevalence in the adult population in Germany remains high (28%) [4]. Moreover, there is a strong link between lower socioeconomic status (SES) and higher prevalence of smoking [4], further increasing health-related inequalities between people of higher and lower socioeconomic groups [5].

Article 14 of the World Health Organization (WHO) Framework Convention on Tobacco Control (FCTC) [6], ratified by Germany in 2004, directs ratifying countries to promote and assist with tobacco cessation. Guidelines for the implementation of Article 14 recommend the integration of brief stop-smoking advice into all health-care systems using existing health infrastructures that are easily accessible to smokers (including primary health care), and ensuring that all health-care workers are trained to provide such advice to their smoking patients [6].

Compared to unassisted attempts to quit tobacco use, brief advice to quit smoking has been found to increase rates and success of attempts to quit smoking [7], and to be effective and affordable [8]. Higher long-term abstinence rates are achieved when using a combination of brief medical advice and evidence-based pharmacological treatment (e.g., nicotine replacement therapy (NRT), varenicline or buproprion) [9, 10]. Hence, national and international clinical smoking cessation guidelines [10–12] recommend that general practitioners (GPs) should routinely give brief quit-smoking advice to every smoking patient and make an offer of help to quit.

Approximately two third of all smokers in Germany report at least one GP visit per year [13]. Hence, appropriate interventions to reduce tobacco consumption in Germany, if implemented in the context of primary care, could reach the majority of the smoking population within a relatively short amount of time.

However, implementation of these guidelines into primary routine care appears to be insufficient. Recent data from a representative household survey on tobacco and e-cigarette use in the German population (DEBRA study, German: “DEutsche Befragung zum RAuchverhalten”, www.debra-study.info) showed that about 80% of smokers who had reported a GP visit during the previous year, had not received advice to quit smoking during this consultation, and barely 4% had been offered an evidence-based cessation method [13]. Comparable data from England showed that about 60% of smokers reported the receipt of such advice [14]. The combination of brief advice to quit smoking with or without a recommendation of pharmacotherapy is offered about seven times more frequently in England [14] than in Germany (25% vs. 4%) [13]. Thus, GPs in Germany are missing a prime opportunity to improve the health of their smoking patients. As a consequence, most smokers (> 60%) in Germany still try to quit smoking unaided or with the use of non-evidence-based treatments [4, 15] and therefore only have a low chance to succeed [16].

Important factors that have been reported as major barriers for GPs in Germany to routinely provide brief smoking cessation advice to their patients include the lack of adequate reimbursement of costs that comes along with the provision of smoking cessation counselling for physicians, reimbursement of patients’ costs for the use of evidence-based smoking cessation treatments, as well as the lack of time during routine consultations [17, 18]. As training in smoking cessation promotion is not a standard part of medical education in Germany, the lack of training or competence in delivering effective advice to quit smoking is another frequently reported major barrier [17–20].

A Cochrane systematic review showed that providing smoking cessation training to health professionals significantly increased their performance in delivering brief behavioural support compared to untrained health professionals, and had a measurable effect on the point prevalence of smoking and on continuous abstinence in patients [21]. However, only few of the included studies were conducted in the context of primary care. In one study conducted in London [22], GPs were offered a 40-min training session addressing the rationale and skills for referral of smokers for cessation treatment. This intervention significantly increased GP referrals to smoking cessation services. A more recent study conducted in primary care in Greece found that a training of 8 hours together with two 3-h refresher trainings significantly increased the rates at which GPs delivered brief stop-smoking advice, evidence-based smoking cessation treatment, as well as self-reported knowledge and self-efficacy in providing such brief counselling at a four-months follow-up [23]. To date only one cluster randomised trial on providing smoking cessation training has been conducted in GP practices in Germany, comparing the effect of a training session together with either physician payment for providing stop-smoking advice or with patient payment for using pharmacological therapy [24]. Hence, no conclusions can be drawn regarding the unique effect of such training in German primary care.

Whereas the majority of studies offered a treatment intensity of at least several hours up to days [21, 23], two recent studies suggest that even short training sessions of one and respectively 3.5-h can significantly increase the GP- and patient-reported performance of delivering brief behavioural support at least in the short- to mid-term [25, 26]. Moreover, the study of Bobak et al. [26] shows that such brief training also leads to substantial and sustained changes in GP trainees’ perceived “**C**apability” (e.g., knowledge/skills) and “**O**pportunity” to provide evidence-based smoking cessation advice [26], reflecting two of the three determinants of actual **B**ehaviour according to the COM-B theory of behaviour [27]. The third determinant “**M**otivation” was high throughout the study [26].

The current national guideline [10] for treating tobacco addiction recommends the delivery of stop-smoking advice according to the so-called “5A” or “ABC” method. Both methods differ regarding content and duration. By tradition, the 5A method is used in Germany consisting of the steps: ask (record and document smoking status of patients), advise (strongly urge all smokers to quit), assess (determine willingness to make a quit attempt), assist (provide evidence-based smoking cessation treatment), and arrange (follow-up contacts) [11]. According to the 5A method, assistance would only be offered to smokers stating their willingness to quit at the time of the consultation (“opt-in” approach). Smokers unwilling to quit, which is true for the majority of smokers [28, 29], should be provided the so-called “5R” approach to enhance motivation to quit (5R includes a discussion with the smoker about the risks of continued smoking, the relevance for and rewards of quitting, potential roadblocks, and the repetition of this approach in subsequent consultations) [11]. Hence, applying all steps of 5A may take 15 min or more and can be difficult to implement during routine consultations. Since the average time slot for GP consultations in Germany is often not longer than 10 min, most patients are unlikely to receive the full 5A protocol: research found the last two steps (i.e., assist, arrange) to be least reported [30, 31], although association with increased quitting is strongest for these two steps of 5A [32]. Furthermore, a considerable number of “unmotivated” smokers may still quit at a later stage [28]. These smokers, according to 5A, would not be offered treatment by their GP and are therefore less likely to use evidence-based treatment to support their quit attempt.

In contrast to this so called “opt-in” approach of 5A [33], the alternative “ABC” method (ask, brief advice, cessation support), which has already been implemented and replaced the 5A method in the New Zealand smoking cessation guidelines [34], is usually applied as an “opt-out” approach [33]. According to this approach, every smoking patient should receive brief advice to quit together with an offer of evidence-based smoking cessation treatment, without assessing and discussing the patient’s current motivation to quit. This approach is based on findings showing that the mere offer of treatment – opportunistically in all smokers, regardless of their current level of motivation – may trigger a quit attempt [35]. Moreover, this method is less time consuming and much simpler to apply than 5A. Hence, it can be assumed that ABC is more convenient to apply at least during routine primary care. However, so far, there is no evidence whether the 5A or the ABC method should be preferred, or whether both are equally effective in increasing rates of delivery of medical advice to quit smoking.

In 2016, our research unit developed and pre-tested 3.5-h-trainings for GPs according to 5A and ABC to improve knowledge on, and practical skills in providing brief smoking cessation advice according to the current national guideline for treating tobacco addiction [10]. During the process evaluation of a pilot study, qualitative data were collected from participating GPs to further optimise the training. Based on this pre-testing, the training concept was finalised. As a strategy for the implementation of the national clinical practice guideline for treating tobacco addiction, both trainings will be evaluated regarding their effectiveness to increase the rates of delivery of brief stop-smoking advice by GPs during routine care.

The present study protocol describes a pragmatic 2-arm cluster randomised controlled trial (cRCT) with a pre-post-design for the primary outcome (evaluation of the effectiveness of a training for GPs on the rate at which they deliver brief stop-smoking advice during routine consultations with smoking patients), and a cluster randomisation for the comparison of the effectiveness of both training methods - 5A and ABC – against each other (secondary outcome).

### Preparatory work

#### Development of the training (intervention), training manuals, and material

In 2016, the following preliminary work was carried out: (a) detailed literature research and professional exchange with national and international colleagues; (b) development of two 3-h-trainings on delivering brief advice to quit smoking according to the 5A and to the ABC method together with experienced GPs working at the Institute of General Practice (ifam) at the Medical Faculty of the Heinrich-Heine-University (HHU) Düsseldorf; (c) preparation of clinical case vignettes of tobacco-dependent patients for simulated role plays with professional actors (standardised patients, SPs) in close collaboration with the CoMeD team (Communication in Medical education) of the HHU; and (d) development and successive adaptation of training manuals. First drafts of patient questionnaires were developed.

#### Pilot study

During the second half of 2016, a pilot study was carried out to test recruitment procedures, content and schedules of the training, practicability of training materials, and methods of data collection.

In total, 14 GPs from 13 practices were recruited in the German province North Rhine of the federal state North Rhine-Westphalia (NRW) by postal dispatch of information material and follow-up phone calls. GPs were randomised to receive training in either ABC or 5A. Four trainings (two on each method respectively) were carried out: 6 GPs participated in the 5A training, and 8 in the ABC training.

All patients consulting their GP during a period of 4 weeks following the training were asked by the GP’s practice nurse, prior to consultation, for their written informed consent to participate in the trial. Consenting patients were asked to fill out a baseline questionnaire on current daily and occasional tobacco use, sociodemographic characteristics, and patients’ contact data. Patients aged 18 years or above who stated to smoke tobacco (cigarettes, hand-rolled or self-stuffed cigarettes, pipe, or cigars) at least occasionally were included in the pilot study.

Within the first week following the consultation with the GP, smoking patients were contacted by telephone by a researcher of the study centre, and were interviewed regarding planned outcomes of the main trial, e.g., on their smoking behaviour, whether or not their GP delivered advice to quit (according to 5A or ABC) during the last consultation, and on their health-related quality of life (EuroQol five dimensions questionnaire (EQ-5D) [36]).

One postal follow-up was conducted 4 weeks later, collecting questionnaire data on further planned secondary outcomes for the main trial (e.g., whether or not patients had made an attempt to quit since the baseline consultation with their GP, or on details about the trigger for and the use of smoking cessation treatment during this attempt (if so)).

This pilot study was approved by the ethics committee of the medical faculty at the HHU Düsseldorf, Germany (5354R).

### Process evaluation, “lessons learned”, and adaptions to the main study protocol

A process evaluation was carried out to gain deeper insights into the feasibility and practicability of the training. To refine the training according to the needs of GPs, problem-centred interviews with GPs of the pilot study were conducted in the first half of 2017. GPs were asked to report their experiences during the training, as well as their perception about facilitating and inhibiting factors for the access and on the transfer of the content of the training into their daily routine.

Interviews were digitally recorded, transcribed verbatim, and analysed using content analysis [37, 38]. Data analysis was conducted computer assisted (MAXQDA). In a multi-professional team (psychology, sociology, public health), core themes were identified to refine the training manual for the cRCT.

Six interviews were conducted with GPs (4 women, 2 men, age range 41 to 61). Participants were included in the qualitative study until saturation of data. The interviews lasted between 21 and 35 min.

#### GPs’ perspective

Balance of theoretical, reflective and practice elements, standardization of role plays owing to professional actors, peer coaching, and formal aspects (e.g., time frame, setting, and date) of the training were positively evaluated and met GPs’ needs for participating in medical education. Motivation to assist patients with quitting tobacco was high following the training, but effects on the rate of successful quitters were expected to be small, reflecting the GPs’ uncertainty about their role in smoking cessation. Peer feedback during role plays was highly appreciated, but SPs’ feedback was perceived as not reflecting real patient-doctor-relationships. GPs reported a need for informational material to pass on to patients.

Based on these results, the training manual was further optimised:

- ➢ Educational objectives were added to the training’s introductory lecture to strengthen GPs’ self-perception as supporter and trigger of quit attempts rather than being responsible for the quit attempt’s success.
- ➢ The duration of training was extended to 3.5 h in order to enable more intensive role-plays with peer feedback. Feedback from SPs was removed from the manual.
- ➢ Additional information material was developed including a leaflet referring to (local) smoking cessation programs to pass on to patients.

#### Study procedures

Based on these “lessons learned” regarding the data collection within GP practices, the following modifications were made to the study protocol and processes of data collection of the main cRCT:

- ➢ Patient recruitment by practice nurses was insufficient and could not be increased substantially by providing financial incentives. Moreover, quality of baseline data was low when patients had to complete the questionnaire by themselves. Hence, in the main cRCT, recruitment of patients and baseline data collection including the primary and several secondary outcomes, will be conducted by researchers of the study centre through face-to-face interviews at the practices. Thus, we aim to yield a maximum response rate, improve the quality of the data, reduce the number of missing values, and minimise recall bias.
- ➢ Following the trainings, GPs seem to have changed their attitude towards the provision of brief stop smoking advice. We therefore decided to collect data on whether the training affects self-perception of GPs’ attitude towards, knowledge on, and practical skills in the provision of brief advice to quit tobacco consumption.

A final evaluation of the training manual and the didactic methods was carried out together with experienced GPs and peer trainers.

## Methods/design main trial

### Objectives

The primary objective is to assess the effectiveness of a 3.5-h-training for GPs in delivering brief advice to quit tobacco consumption, irrespective of the training method (5A or ABC), on patient-reported rates of delivery of such advice by their GPs during routine consultations in a German primary care setting.

### Secondary objectives are

- to assess the effectiveness of the training, irrespective of the training method, on patient-reported rates of delivery of recommendations or prescriptions of evidence-based smoking cessation treatments to assist their quit attempt: (secondary objective (S1) behavioural treatment (single or group intervention), (S2) NRT, and (S3) varenicline or bupropion;
- to assess the effectiveness of the training, irrespective of the training method, on patient-reported quit attempts (S4–6) and point-prevalence abstinence rates (S7–9) at 4, 12, and 26 weeks after GP consultation;
- to directly compare the effectiveness of the two methods (5A vs. ABC) by means of the primary outcome and the secondary outcomes S1–9 (S10).

### Study design

The finalised training will be evaluated in a pragmatic 2-arm cRCT with a pre-post-design (pre-training = care as usual) for the primary outcome, and a cluster randomisation for secondary outcome (comparison of the effectiveness of both training methods - 5A and ABC – against each other) in approximately 48 GP practices (cluster) in NRW (Germany) between June 2017 and February 2020. The intervention will be a 3.5-h-training for GPs in providing brief stop-smoking advice according to the 5A or ABC method. Data on primary and on several secondary outcomes will be collected prior to and following the training by interviewing consecutive patients of participating GPs. Further secondary data will be collected by means of three follow-up questionnaires.

**Fig. 1:**
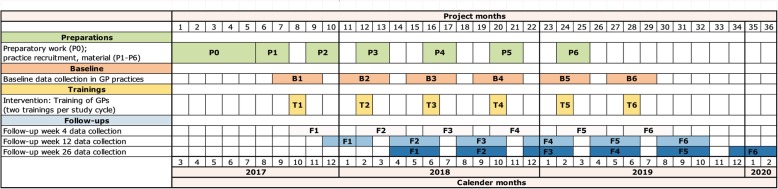
Gantt chart of the study illustrating the schedule of the total study, including the preparation period, pilot testing of the intervention, the distribution of the six study cycles (intervention) over the total duration of the study, and all periods of data collection. P0 Process evaluation pilot study, finalisation of training and manual, ethical approval, study registration. P1-P6 Preparations study cycles 1 to 6: practice recruitment, material, application for CME certification, scheduling of trainings. B1-B6 Pre- (7 days) and post-baseline (7 days) data collection of primary and secondary outcomes (S1-S3) in 8 GP practices per study cycle. T1-T6 Intervention: 3.5-h training of GPs in delivering brief stop-smoking advice according to either 5A or ABC. F1-F6 Postal follow-up data collection of secondary outcomes (S4-S9) corresponding to study cycle 1–6

In total, there will be six study cycles with 8 participating GP practices per cycle. A study cycle is defined as a period of 8 weeks with the training of GPs from four practices in the 5A method and GPs from four practices in the ABC method in the middle of this period. Data collection will be carried out on approximately seven varying GP office days during the 4-week pre-training period and on 7 days during the 4-week post-training period. Thus, we aim to minimise sampling bias which could result from considerable fluctuations in patient flow among different days or weeks of data collection (e.g., on Mondays, during flu epidemic, or on holidays). The distribution of study cycles over the total duration of the study is shown in the Gantt chart of the study (Fig. 1).

### Setting and sample (participants)

**Fig. 2:**
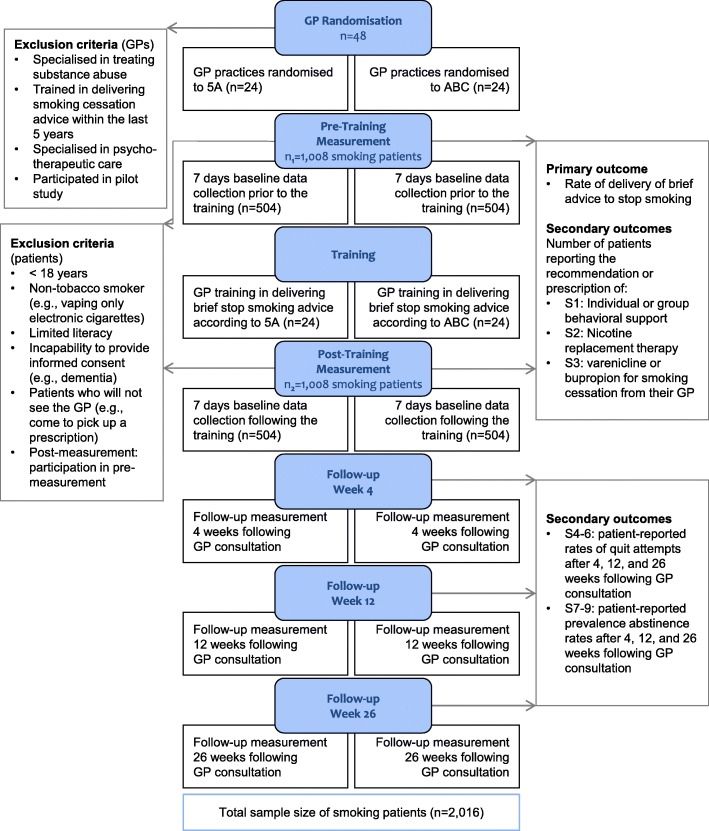
Consolidated Standards of Reporting Trials (CONSORT) flow chart illustrating all steps in the study from enrolment to training allocation and follow-ups. Inclusion and exclusion criteria are also specified as well as outcome measures

The study will be coordinated at the ifam of the HHU Düsseldorf. Figure 2 shows the flow chart of the study according to the Consolidated Standards of Reporting Trials (CONSORT).

### Recruitment, eligibility, and randomisation

#### GP practices

GP practices will be mainly recruited from the publicly accessible online medical register of the regional Association of Statutory Health Insurance Physicians North Rhine, and also from the shared GP practice network of the institutes of general practice of the HHU Düsseldorf and the University Witten/Herdecke. GPs will be informed about the study and training dates, and invited to participate by postal dispatch of study information and follow-up phone calls. All GPs will be eligible for participation, except for those specialised in treating substance abuse or in psychotherapeutic care, or those who have already been trained in providing smoking cessation support within the last 5 years, including GPs who participated in the pilot study. Participating GPs will have to give their consent to take part in a 3.5-h-training in delivering brief stop-smoking advice and to support a 4-week phase of data collection in their practice prior to and following the training.

Per study cycle, three to four potential training dates will be offered. GPs will be asked to register at least for two dates. Depending on the number of registrations per training date, two different methods of randomisation will be applied in each cycle:

A) If a sufficient number of GPs (> 8) will be available at least at two of the proposed dates, computer-generated block randomisation will be applied, using random permuted blocks of sizes 2 or 4. The randomisation sequence will be prepared in advance by an independent statistician (WV) and will be concealed from the researchers at the study centre who are involved with the conduct of the trial. The same method can be applied, if eight or more GPs will register on the same date. Both trainings (5A and ABC) will then be carried out on the same day.
B) If fewer than eight GPs will be available at two of the proposed dates, GPs will be randomised “naturally” by virtue of the GPs temporal availability. Two dates with most of the registrations will be selected. GPs registered at the first date will receive training according to the 5A method. GPs registered at the second date will receive training according to the ABC method. This order will be alternated between cycles.

In order to prevent contamination, group practices with more than one participating GP will be assigned to the same study arm, depending on the allocation of the GP from this group practice who is assigned first. If only some of the GPs of a group practice participate in the study, only patients of assigned GPs will be included in the study. In case a GP has to cancel his or her participation in the training in the short-term, but pre-training data collection was successfully completed (e.g., in study cycle 3), this GP will be trained in the subsequent study cycle (e.g., study cycle 4) to prevent drop-out of this GP. Post-training data in his or her practice will then be collected during the same study cycle in which the training occurred (e.g., study cycle 4).

#### Patients

Patients 18 years or older visiting participating GPs during a study cycle will be asked for to provide informed consent to participate in the study. Patients suffering from moderate or severe cognitive impairment, or those of too low literacy, will be excluded. Moreover, patients who will not talk to their GP in person will be excluded (e.g., patients who just come to pick up a prescription or to escort a relative).

The study team involved in the data collection consists of four part-time researchers (Bachelor/Masters’ degree in psychology, public health, and health economics). Each researcher can manage data collection in two of the eight GP practices in each study cycle. Prior to data collection, the researchers will inform nurses in participating GP practices about the study schedule, and ask for assistance regarding patients who do not meet the inclusion criteria. Posters and leaflets promoting the study will be provided to the practices.

During a data collection day in the practice, patients will be informed about, and invited to participate in the study by the researcher in the waiting room, prior to their consultation with the GP. Consenting patients will be asked to participate in a brief interview immediately following this consultation. At this time, patients will not be fully informed about the real purpose of the study, because addressing the topic of smoking at this time could trigger patients to initiate a discussion about their smoking during the consultation. Hence, prior to the consultation, patients will be asked to participate in a study on “physician-patient communication on health behaviour”. This strategy has been approved by the ethics committee.

Brief interviews (~ 10–15 min) to collect baseline data (see Additional file 1, translated versions are also provided) will be conducted consecutively with every single patient. All patients will be asked on sociodemographic characteristics (e.g., age, sex, level of education), health-related quality of life, and on smoking status. Never and ex-smokers will receive full information on the purpose of the study and the interview will be interrupted at this moment. Patients who only use electronic cigarettes or heat-not-burn products but who do not smoke tobacco cigarettes will also be excluded at this moment. Furthermore, those patients will not be included in any follow-up data collection.

Current daily and non-daily tobacco smokers (cigarettes, cigarillos hand-rolled cigarettes, pipe, cigar, or hookah) will be further interviewed on their smoking behaviour, motivation to stop smoking [28], strength of urges to smoke [39], as well as on primary and secondary outcomes. At the end of this interview, patients will be informed about the purpose of the misinformation regarding the interview content according to the requirements of the ethics committee.

By assessing the above-mentioned primary and secondary outcomes immediately after GP consultation through a face-to-face interview, we aim to maximise response rates and minimise recall bias.

### Blinding

Due to the pragmatic nature of the study design it will not be possible to fully blind GPs to their training allocation (5A or ABC method). However, to minimise possible systematic bias, where GPs tend to make more effort in delivering brief advices to quit depending on the training method they were allocated to, we aim to reduce the information on the group allocation until the end of the 4-week pre-training data collection period. In particular, during the recruitment process and during the pre-training period, GPs will only be informed that the implementation of the brief advice in GP practices is being examined and that the effect of two different brief advice methods is compared. The GPs, however, received no information about the content and the difference of both methods.

Initially, we planned to blind the researchers conducting the baseline data collection to the GPs’ group allocation. However, with eight GPs per study cycle and four part-time researchers collecting data in their practices, it will not be feasible to conceal group allocation to the study team. In order to prevent that GPs behave differently depending on the person collecting data in their practice, these researchers will not be actively involved in the training of the GPs.

### Intervention

Depending on their group allocation, GPs will receive training on the delivery of brief stop-smoking advice either according to the 5A or to the ABC method. Both types of training will be conducted in accordance with the national and international guidelines on smoking cessation [10, 11]. In both groups, the training will be organised at a central location (i.e., the HHU in Düsseldorf), and will have a duration of approximately 3.5 h starting at a time which is feasible and familiar to GPs (late afternoon/early evening on Wednesdays and Fridays, or on Saturdays). In order to avoid seasons of high workload, and to avoid a low reach of patients during holiday periods, study cycles will primarily be carried out during the months February/March 2018/2019, September/October 2017/2018, and May/June 2018/2019 **(**Fig. 1**)**.

Both types of training will include an introductory lecture of approximately 60 min about tobacco addiction, evidence-based smoking cessation treatments, and about the specific method of stop-smoking advice (either 5A or ABC). Reflexive units and discussions on GPs’ experience with the provision of stop-smoking advice, and on associated barriers and facilitators, will also be a part of the introductory lecture.

This lecture will be followed by an intensive role play of about 90–120 min with professional actors trained in patients’ specific behaviour. In these role plays, GPs get to practise the respective training method and receive moderated feedback (see below for further details regarding the content). Training sessions will always be led by a pair of trainers: a senior researcher of the study centre and an experienced GP (peer-trainer) who can specifically address the questions and needs of daily GP routine. For both types of training, the number of participants will be restricted to a minimum of three and a maximum of ten (in case of participating group practices). A detailed manual has been developed which the trainers should refer to. Thus, we aim to standardise the contents, schedule, and quality of each training session as much as possible. In order to avoid that the quality of training sessions differs between 5A and ABC, and might thus impact the study outcome, we will rotate the pairs of trainers from study cycle to study cycle. Face validity of the training was assessed during the pilot study and will be assessed again during a test run prior to the start of the main trial, where trainers, actors, and two practicing GPs will rehearse and review the clinical vignettes for the role plays which have been planned in close collaboration with the CoMeD at the HHU Düsseldorf.

The CoMeD team is engaged in the training of professional actors to become an SP and learn how to give constructive feedback for simulated role plays in medical communication education. Different types of, for example tobacco addicted, patients can be portrayed by one SP. These SP trainings are a major element in order to guarantee high quality education of patient-physician-communication [40].

Both trainings will teach GPs how to ask patients about smoking (A1) and how to advise them to stop (A2). The 5A training will specifically teach GPs how to: assess the patients’ willingness to quit (A3) and how to apply the 5R approach in patients who are not willing to quit; assist the quit attempt (A4); and arrange follow-up (A5). The ABC training will specifically teach GPs how to make the offer of treatment (A4) opportunistically (e.g., a prescription for NRT, or a referral to individual or group behavioural support; irrespective of the patients’ motivation to stop at the time of the consultation). In addition to the training, GPs from both intervention groups will receive paper-based leaflets summarising the key aspects of 5A or ABC, a one-page handout with short information on evidence-based smoking cessation treatments which can be offered to patients, as well as a one-page copy template with several local outpatient programs to which smoking patients can be referred to. The completion of the training, irrespectively of the method, will be incentivised with Continuing Medical Education (CME) credits. Material for GPs of both groups will be of exactly the same quality and adhere with common standards for CME.

Considering the above, it can be assumed that the 5A training would require more time than the ABC training. However, during the pilot study, we noticed that teaching the ABC method demands extra time for group discussions on offering smoking cessation treatment irrespective of patients’ motivation to stop, as GPs seem less familiar with this opportunistic approach. Hence, duration was kept the same for both trainings.

### Theoretical foundation of the intervention: COM-B model

The theoretical foundation of the intervention is based on the “COM-B” model developed by Michie and colleagues [27]. According to this model, the interaction between the three components capability (C), opportunity (O), and motivation (M) causes the performance of behaviour (B). Each component influences behaviour and, moreover, capability and opportunity influence motivation and therefore affect behaviour. COM-B is a dynamic model whereby performance of a specific behaviour can in turn influence capability, opportunity, and motivation. Interventions need to change one or more of the components in such a way as to put the system into a new configuration. Interventions need to change one or more of the components in such a way as to achieve a desired change in behaviour.

According to studies which explored barriers to the promotion of smoking cessation in German primary care, GPs often lack the feeling of competence in giving cessation advice (capability: knowledge, practical skills) [17–19], inadequate reimbursement of costs (opportunity) involved in the promotion of smoking cessation and, additionally, rate these conversations to be too time consuming (capability, motivation) [17, 41]. In our trainings, we aim to address at least these two components (capability and motivation) of the COM-B Model and expect that this might affect the GPs’ behaviour of offering brief stop-smoking advice to their smoking patients during routine consultation. Moreover, it can be hypothesised that giving brief stop-smoking advice according to ABC might influence the component opportunity because this method is less difficult and less time consuming to apply, and can be integrated more easily into the daily practice routine.

For a better description of the main active elements of the developed trainings, we aim to use the Behaviour Change Techniques (BCT) Taxonomy from Michie and colleagues [42]. A training session can be categorised in three superordinate elements: (1) introductory lecture, (2) practice element with role plays, and (3) reflexive elements. Reflexive elements, however, also occur in group discussions during the role plays or following the introductory lecture. BCTs that will be applied during each training session are listed in Table 1.

**Table 1:** Exemplary contents of the ABC and 5A training with corresponding codes and labels according to the Behavior Change Technique (BCT) Taxonomy^a^

Superordinate elements of training	BCT group^a^	BCT code and label^a^	Examples from the training or training manual
1 Introductory lecture	1 Goals and planning	1.2 Problem solving	Prompt GPs to identify barriers preventing them from starting a conversation on smoking cessation during routine consultations and discuss potential solutions. For example how to advise patients with multiple unsuccessful quit attempts in their history, how to start a conversation on smoking cessation with patients having no smoking-related problems.
	4 Shaping knowledge	4.1 Instruction how to perform the behaviour	Inform GPs verbally on how to provide brief advice to stop-smoking according to 5A/ABC with examples of different types of patients.
	5 Natural consequences	5.1 Information about health consequences	Inform GPs verbally on health risks of smoking and benefits of smoking cessation, and on the role of GPs in reducing smoking prevalence on a population level.
		5.6 Information about emotional consequences	Pointing out that the provision of brief-stop smoking advice aims at triggering a quit attempt rather than long-term abstinence in every smoker receiving such an advice. Lowering high or delusive expectation should lead to a reduction of frustration, and thus increase self-efficacy. Only ABC training: Provide information on how application of the ABC method to deliver brief-stop smoking advice (without discussing patients’ motivation to quit) can reduce stress and frustration in daily GP routine, and thus increase satisfaction.
	6 Comparison of behaviour	6.1 Demonstration of the behaviour	Demonstrate to GPs how to raise the issue of smoking cessation with patients indirectly via pictures of exemplary patient-physician conversations.
		6.2 Social comparison	Providing information on the proportion of smokers in Germany who were offered GP advice on quitting by their GP, thus they can compare with their own performance.
		6.3 Information about others’ approval	Telling GPs that smoking patients will appreciate a conversation on smoking cessation including the provision of support/assistance rather than a conversation with criticism or reproaches causing feelings of guilt in patients.
	7 Associations	7.1 Prompts and cues	Provision of handouts for GP practice rooms to remind them of delivering brief stop-smoking advice to all smoking patients: including: the 5A/ABC method, behaviour change techniques for patients (e.g. setting a quit day), the Fagerström Test for Cigarette Dependence^b^, information on evidence-based smoking-cessation therapy, contact information: smokers’ telephone helpline, regional group-based smoking cessation programs).
	8 Repetition and substitution	8.2 Behaviour substitution	ABC: Suggest that GPs should not ask for patients’ motivation to stop smoking and provide their assistance instead to every smoking patient regardless of motivational status. 5A/ABC: Suggest that GPs provide stop smoking support as a brief or very brief conversation on smoking cessation rather than as a time consuming and exhausting discussion.
	9 Comparison of outcomes	9.1 Credible source	Presentation of evidence-based data (e.g., data from Cochrane reviews) on the importance and effectiveness of brief GP advice to stop-smoking.
	11 Regulation	11.2 Reduce negative emotions	Advise GPs how to reduce frustration during stop smoking conversations (e.g., through realistic goal setting: aiming to trigger a quit attempt rather than long-term abstinence in smoking patients).
	13 Identity	13.2 Framing/reframing	Cognitive structuring: Suggest that medical advice on quit smoking must not necessarily be time consuming and exhausting (which are frequently reported barriers preventing GPs from raising a stop-smoking conversation).
2 Practice elements (Role plays with peer feedback)	1 Goals and planning	1.1 Goal setting (behaviour)	GPs are encouraged to apply all the steps of ABC/5A during the role play and therefore change their familiar patterns of behaviour during conversations on smoking cessation.
		1.2 Problem solving	Discussions during role plays: prompt GPs to identify barriers preventing them from applying a specific step of 5A or ABC (“Which steps of 5A or ABC could you (not) apply during the role play, and why or why not?” “What could have helped/could be changed during in this situation?”).
		1.5 Review behaviour goal(s)	Examine how well a GP’s performance during role play corresponds to agreed goals (e.g., applying all steps of the 5A/ABC method, or providing brief advice on quitting to the patient without reproaches or criticism); and consider a modification of a behavioural goal, e.g. through realistic goal setting: aiming to trigger a quit attempt rather than being responsible for the quit attempt’s success.
		1.6 Discrepancy between current behaviour and goal	Trigger a quit attempt rather than long-term abstinence in smoking patients; Trainer and peers point out and discuss which steps of 5A or ABC had not been applied during role play.
	2 Feedback and monitoring	2.2 Feedback on behaviour 2.7 Feedback on outcomes of behaviour	Peers and trainers provide moderated feedback on GP’s behaviour/ performance and on observed outcomes (reactions) of patient (actor) during role plays.
	3 Social support	3.2 practical support 3.3 emotional support	Peers and trainers provide practical and emotional support during role plays: e.g., advise on how to cope with a specific patient reaction.
	4 Shaping knowledge	4.1 Instruction how to perform the behaviour	Repeated instructions (verbal) are provided by the trainers prior to the role plays: how to provide brief advice to stop-smoking according to 5A/ABC.
	5 Natural consequences	5.4 Monitoring of emotional consequences	GPs are encouraged to reflect and reveal their feelings during active role play.
	6 Comparison of behaviour	6.1 Demonstration of the behaviour	Provision of role plays with moderated feedback to practice the delivery of brief stop-smoking advice according to 5A/ABC.
		6.2 Social comparison	GPs are encouraged to observe the performance of colleagues during role play allowing comparisons with their own performance during role play but also during past routine practice consultation.
		6.3 Information about others’ approval	Peers and trainers provide feedback on the performance of the GP who participates in the role play.
	8 Repetition and substitution	8.1 Behavioural practice/rehearsal	Provision of role plays with moderated feedback to practice the delivery of brief stop-smoking advice according to 5A/ABC.
		8.2 Behaviour substitution	Trainer and peers suggest alternative reactions/sentences during role plays corresponding to the 5A/ABC method (e.g., ABC: providing assistance with attempt to quit rather than discussing patients’ motivation to quit smoking).
	9 Comparison of outcomes	9.1 Credible source	GP peer trainer reports on own positive experiences but also on challenges with the provision of brief stop-smoking advice according to either the 5A or ABC method.
	13 Identity	13.2 Framing/reframing	Providing measurements on the exact duration (minutes) of role-play in order to demonstrate that the provision of brief advice on quit smoking must not necessarily be time consuming, which is a frequently reported barrier preventing GPs from raising a stop-smoking conversation.
3 Reflexive elements (Group discussions at the beginning and end of the training)	1 Goals and planning	1.1 Goal setting (behaviour)	Prompt GPs to set a self-defined goal for the next working day regarding the provision of brief stop-smoking advice with the so-called “Monday-Question”: “What would you change/ do differently next Monday in practice?”.
	9 Comparison of outcomes	9.2 Pros and cons	Encouraging GPs to reflect the advantages and disadvantages of providing brief stop-smoking advice (more often) to their smoking patients.
	13 Identity	13.3 Incompatible beliefs	Drawing attention to discrepancies between GPs’ current or past performance regarding the provision of advice to quit smoking and his or her self-image as a health consultant.
	15 Self-belief	15.3 Focus on past success	Encourage GPs to reflect strategies which helped them in the past to have a successful conversation on smoking cessation with a patient.

^a^Taken from Michie, S., Richardson, M., Johnston, M., Abraham, C., Francis, J., Hardeman, W., Eccles, M. P., Cane, J. & Wood, C. E. (2013). The Behavior Change Technique Taxonomy (v1) of 93 Hierarchically Clustered Techniques: Building an International Consensus for the Reporting of Behavior Change Interventions. Annals of Behavioral Medicine, 46(1), pp. 81–95. doi: 10.1007/s12160-013-9486-6. Available from: https://link.springer.com/article/10.1007/s12160-013-9486-6. (Accessed 30.07.2018)

^b^Fagerström, K. Determinants of Tobacco Use and Renaming the FTND to the Fagerström Test for Cigarette Dependence Nicotine Tob Res (2012) 14 (1): 75–78

### Outcomes

#### Primary outcome

The primary outcome is defined as the number of patients who report the receipt of brief stop-smoking advice during the last consultation with their GP, irrespective of the training method, out of the total number of patients who stated to be current tobacco smokers at the time of the consultation with their GP. These data will be collected prior to and following the training through personal interviews in all consecutive patients immediately following GP consultation.

#### Secondary outcomes

Secondary outcomes S1–3 will be collected together with the primary outcome through personal interviews immediately after GP consultation and refer to the patients’ last consultation with their GP. Data on secondary outcomes S4–9 will be collected by means of three follow-up questionnaires by postal dispatch. The denominator of all secondary outcomes is the number of patients who stated to be current tobacco smokers at the time of the consultation with their GP.

- S1: Number of patients who report the receipt of a recommendation for individual or group behavioural support for smoking cessation;
- S2: Number of patients who report the receipt of a recommendation or prescription of NRT for smoking cessation;
- S3: Number of patients who report the receipt of a recommendation or prescription for varenicline or bupropion for smoking cessation;
- S4–6: patient-reported rates of quit attempts after 4, 12, and 26 weeks following the consultation with the GP;
- S7–9: patient-reported point prevalence abstinence rates at 4, 12, and 26 weeks following the consultation with the GP;
- S10: Interaction effect between time (from pre-to post-training) and group variable (5A vs. ABC) for the primary and secondary outcomes S1-S9.

Furthermore, this study aims to assess additional data in GPs on to what extent one 3.5-h-training session according to either the 5A or the ABC method affects, at least in the very short-term (immediately following the training), self-perception of GPs’ attitude towards (motivation), knowledge on, and practical skills (capability) in the provision of brief advice to quit tobacco consumption.

### Data collection

#### General practitioners

A brief questionnaire will be administered to GPs prior to the training to collect information on the characteristics of the participating GPs (including age, gender, smoking status, professional experience, and specialisation) and the practice characteristic, such as location (rural vs. urban), average number of patients per calendar quarter.

Within the 4 weeks prior to and immediately following the training, GPs will be asked to fill out a brief questionnaire to collect data on their attitude towards (motivation), knowledge on, and practical skills (capability) to provide brief stop-smoking advice during routine consultations. In this questionnaire, GPs will be asked to indicate whether they are aware of the steps of the designated method, whether they provide brief stop-smoking advice regularly or not, whether they think that providing brief advice to their smoking patients is important, effective, and feasible in GP practices, and whether they are confident in providing such advice, respectively according to either 5A or ABC. Answering options will be: (1) “strongly support”, (2) “support”, (3) “tend to support”, (4) “oppose”, (5) “strongly oppose”.

### Patients

#### Baseline measurements

Following the consultation with their GP, a researcher of the study team will immediately conduct the brief baseline interview (~ 10–15 min) with the study (see Additional files 1 and 2). At the beginning of the interview, all patients will be asked about their age, sex, level of education, employment status, and health-related quality of life (EQ-5D [36]).

Patients will then be asked whether they smoke tobacco (cigarettes, hand-rolled cigarettes, pipe, cigar, or hookah). Answer categories will be: (1) “Yes, I currently smoke on a daily” or (2) “on a non-daily basis”, (3) “No, but I used to smoke in the past”, (4) “No, I have never smoked daily or occasionally”.

Current smokers of tobacco products will be asked further details on their smoking behaviour: how long it has been since they had smoked their last cigarette, the average number of cigarettes (or, e.g., pipes, cigars) they smoke per day, week or month (for occasional smokers), on their motivation to stop smoking (German version of the Motivation to Stop Smoking Scale, MTSS [28]), and their strength of urges to smoke (German version of the Strength of Urges to Smoke Scale, SUTS) [39]. These variables represent important co-variates or confounders for the planned statistical analyses.

Subsequently, data on the primary outcome will be collected. Smoking patients will be asked whether their GP started a conversation on the patient’s smoking behaviour during the preceding consultation at the same day. If the answer is “yes”, these patients will be asked whether or not their GP provided brief advice to quit smoking according to either 5A or ABC during the preceding consultation. This is operationalised by whether the GP asked for the smoking status (A1: both 5A and ABC), urged the smoker to quit (A2: both 5A and ABC), asked for the patients’ motivation to quit smoking (A3: only 5A), provided evidence-based stop-smoking therapy (A4: both 5A and ABC) by recommending or prescribing either: individual or group behavioural support (S1), NRT (S2), varenicline or bupropion (S3); and arranged a follow-up appointment (A5: only 5A). Furthermore, recommendations of a combination of therapies will be asked (e.g., “my GP recommended or prescribed me individual or group behavioural support AND NRT).

Patients reporting the receipt of any brief smoking cessation counselling will be asked about their satisfaction with that conversation; operationalised by ratings on a 6-point Likert scale ranging from 1 = “very satisfied” to 6 = “very dissatisfied”).

#### Follow-up measurements

Four (time window = + 1 week), 12, and 26 weeks (time windows = + 2 weeks, respectively) after consultation with their GP, patients who reported to be a tobacco smoker at baseline will receive a follow-up questionnaire (see Additional files 3 and 4) by postal dispatch. This questionnaire collects data on secondary outcome (S4-S9): whether or not patients have made an attempt to quit from baseline (GP consultation) to follow-up, and on their current smoking status (S 7-9= point prevalence abstinence rates). In relation to this, questions will be asked about what triggered that attempt (e.g., advice of GP, smoking-related disease, cigarette package health warnings), whether it was supported by evidence-based (e.g., NRT) or non-evidence-based smoking cessation treatments (e.g., acupuncture, hypnosis), and how long this attempt to stop did last (if so). In order to avoid overlaps with further consultations or multiple quit attempts which might have taken place since the initial consultation, the questions will be clearly formulated with reference to specific dates or periods. To assess changes within the health-related quality of life from consultation to follow-up, a paper-based version of the EQ-5D will be sent along with each follow-up questionnaire.

Patients will receive a small unconditional and non-financial incentive together with each follow-up, which is an evidence-based strategy to increase the response rates [43–45].

### Power and sample size

Based on recent data from a study of the German population [13], we assume that about 18% of smokers are currently receiving brief advice on smoking cessation during a consultation with their GP. Training in either the 5A or ABC method is assumed to have a clinically relevant effect if it increases these rates by at least 10% (corresponding to an odds ratio of 1.77) between pre- and post-training.

Calculation of the required sample size is based on the primary analysis and is intended to ensure that a significant time effect between pre- and post-training can be detected with a probability of 80%, taking into account the clustering of patients in GP practices. The following assumptions were made: (a) 48 GP practices will be recruited (six study cycles with 4 GPs receiving the 5A training and 4 GPs receiving the ABC training each cycle); (b) the delivery rate of brief cessation advice prior to the training is 18%; (c) these rates vary among practices with assumed ranges from 9 to 32% in 95% of the practices (corresponding to a standard deviation (SD) of 0.40 on the logit scale); (d) the chance of receiving stop-smoking advice increases on average from 18 to 23% (5A method) or 33% (ABC method) after a training; and (e) varies among practices after the training (between 12 and 40% (5A method) or between 18 and 52% (ABC method) in 95% of the practices (equivalent to a SD of 0.05 for the time effect on the logit scale).

A simulation study based on these assumptions showed that 16 patients (respectively 8 prior to and 8 following the training) per practice are needed to evaluate the primary outcome with a statistical power of at least 80%. A total of 42 patients per practice (21 before and 21 after training) are needed to yield the same power when analysing the interaction between the time and group variable (secondary outcomes S10), resulting in a total study sample size of 2016 patients (respectively 1008 prior to and 1008 following the training). The R-code of this simulation can be provided on request.

Patient participation rates are estimated from data of an earlier study on smoking cessation interventions in GP practices in Germany [46] and on current prevalence rates of smoking in Germany at the moment of developing the study [3]: The number of patients visiting their GP per day is ~ 19, about 28% (*n* = ~ 5) of them are smokers; about 60% of these smokers (*n* = ~ 3) meet the inclusion criteria and are willing to participate in the short interview following the consultation with their GP (primary outcome and secondary outcomes S1-S3). Per study cycle, data collection will be carried out on 7 working days during the 4-week pre-training period and on full 7 working days during the 4-week post-training period, resulting in data from 21 smoking patients prior and 21 smoking patients following the training in each practice (~ 42 smoking patients per practice).

### Planned statistical analyses

#### Descriptive statistics

Demographic data of patients (e.g., age, gender, education, employment status, smoking characteristics) and data of GPs (e.g., age, gender, smoking status, practice characteristic) will be described for the total group and for the study arms separately. Continuous variables will be presented with means, and SD. Categorical variables will be denoted in numbers and percentages. Differences in sociodemographic or practice characteristics among patients and GPs of the two study arms will be analysed using Student t-tests, or non-parametric Mann-Whitney *U* tests. For categorical variables, Pearson χ2 or Fisher’s exact test will be used.

#### Analyses of primary and secondary outcome measures

The primary endpoint is dichotomous: patient reported receipt of brief stop-smoking advice delivered by the GP during the preceding consultation (yes or no). Hence, analyses of the primary outcome will be conducted using logistic regression models. Data are structured hierarchically (in clusters = practices), with the patients located within the practices. Since differences in rates of delivery of smoking cessation advice are expected among the practices, mixed effect models will be used. For the primary analysis, the model contains a fixed effect for time (dichotomous: pre- versus post training) and random effects for the practices and the time effect. This model will be adjusted for potential confounders measured at baseline: age, sex, SES, motivation to stop smoking, and strength of urges to smoke of the patients.

The same model will be applied to the secondary outcomes S1-S3 (recommendation of behavioural treatment, NRT, or medication), S4-S6 (patient-reported quit attempts at 4, 12, and 26 weeks following the consultation with the GP), and S7-S9 (patient-reported abstinence at 4, 12, and 26 weeks following the consultation with the GP).

In order to analyse differences between the 5A and ABC training (secondary outcome S10), the group variable (dichotomous: 5A or ABC training) and its interaction with time will be added to the models as fixed effects. In both models, the time effect and the interaction will be analysed by means of Wald tests (level of significance .05).

All participating patients will be included in an intention-to-treat analysis. The risk for missing data on the primary outcome and secondary outcomes S1-S3 will be relatively small, as these data will be collected though personal interviews. However, missing data will be imputed and recoded as patient-reported “no advice-to quit delivered by GP” (primary outcome) and “no treatment recommended or delivered by GP” (S1-S3). To examine the sensitivity of the results, a complete case analysis will be performed additionally, which means that all cases with missing data on the primary outcome will be excluded from the analysis. Missing data on secondary outcomes S4-S6 will be imputed and recoded as “no quit attempt”. Missing data on secondary outcomes S7-S9 will be imputed and recoded as “smoker” at follow-up.

#### Trial status and timescale

As of January 2019 (revision of the manuscript), the recruitment of GPs and patients had started but had not yet been completed. Data collection including all follow-ups is expected to be completed in February 2020 (Fig. 1).

## Discussion

Given the high number of patients regularly consulting their GP in Germany, primary health care represents the optimum setting for the identification of smokers and the provision of evidence-based smoking cessation treatment to take place. This is addressed in the national guideline for treating tobacco addiction [10], but implementation of this guidance in clinical practice is inadequate. Hence, there is an urgent need for strategies that can improve the implementation of this guideline in the primary health care setting.

Central factors which have been identified as barriers to the promotion of advice on smoking cessation comprise the lack of training and lack of time to provide appropriate advice during routine consultations [17–19]. Interventions aiming to improve the guideline implementation should therefore be as little time consuming as possible to internalise and to apply, and have the potential for a broad implementation in clinical practice. However, most primary care studies so far evaluated trainings with an intensity of several hours up to days, sometimes in combination with refresher trainings [21, 23]. Only a few recent studies suggest that even short training sessions seems to have an effect on the performance of GPs in delivering brief behavioural support [25, 26]. We therefore developed two 3.5-h-trainings as a strategy for the implementation of the national clinical practice guideline for treating tobacco. Both trainings aim to improve at least two of the three determinants (capability and motivation of GPs to deliver brief stop-smoking advice) causing the performance of behaviour according to the COM-B theory [27].

Due to a lack of evidence, the national guideline [10] cannot recommend whether the 5A or the ABC method to deliver brief medical advice to quit smoking should be preferred, or whether both are equally effective. Since ABC, as an “opt-out” approach, seems to be less time consuming and less frustrating to GPs, we assume it to be more convenient to apply at least during routine primary care. Evidence regarding this hypothesis comes from studies showing that the majority of physicians delivers only the first three 5A steps (“ask, advice, assess”) [30, 32], although “assist” and “arrange” are those steps associated with increased quitting [32].

The present study might help to develop a strategy to improve the implementation of brief stop-smoking advice in German primary care. The findings might also help fill a knowledge gap on the effectiveness of two different methodological approaches, the “opt-in” (5A) versus the “opt-out” (ABC) approach to provide brief advice to quit smoking. Moreover, data of this study will give further insight on processing data, e.g., barriers and facilitators for the implementation of the clinical guideline on the treatment of tobacco addiction into clinical practice.

### Strengths and limitations

This study is the first to evaluate the effectiveness of a relatively brief training intervention according to the 5A method in contrast to the ABC method to deliver brief stop-smoking advice during routine consultation in GP practices in Germany. Effectiveness will be measured by means of patient reports. Role plays with professional actors trained in portraying different types of smoking patients as standardised as possible are an essential element of both developed trainings. It has been shown that such role plays with trained SPs are one principal element in order to guarantee high quality education of patient-physician-communication [40].

The randomised controlled study design will reduce the risk of selection as well as interpretation bias. In this study, data on the primary and several relevant secondary outcomes will be collected in patients immediately following the consultation with the GP minimising the risk of missing data as well as the risk of recall bias. The pragmatic approach with relatively unselected participants and under real-practice conditions strengthens the external validity of the results and may provide methodological and processing data for subsequent implementation studies. Moreover, if the training is proven to be effective, its briefness might further facilitate a broad implementation in primary health care.

However, several challenges associated with the pragmatic nature of this study need to be addressed. First of all, as with most interventional studies in primary care, a bias on the selection of GPs is expected, with higher participation rates of GPs with interest in the topic of smoking cessation support and with the possibility or motivation to participate in further training activities. This effect may be assumed to be equally distributed over the study arms. If our hypothesis can be confirmed, the offer of individual GP trainings held at the practice sites might be one solution to allow the implementation of the intervention also in practices with less motivated GPs or with those without the opportunity to take part in group trainings due to massive workload or other structural or organizational barriers.

The presence of a researcher of the study centre for the purpose of data collection could trigger and increase the performance of GPs, and thus lead to an overestimation of the patient-reported rates of brief stop-smoking advice delivered by their GP. However, this is not expected to distort the main findings of the study since the primary outcome will be evaluated by means of the relative difference between pre- and post-training measurements. In contrast, face-to-face interviews with patients assessing smoking-related outcomes, even if such conversations cannot be formally seen as smoking cessation counselling, might act as a potential trigger for subsequent quit attempts (secondary outcome) which cannot be completely prevented. Nevertheless, researchers conducting these interviews will be obliged to refrain from providing any counselling on smoking cessation, and since secondary outcomes will also be evaluated by means of the relative difference between pre- and post-training measurements, we could at most expect to find a ceiling effect regarding this outcome.

Due to the pragmatic nature of this study, GPs and researchers cannot be blinded to the allocation of both study arms. In order to reduce contamination, we recommend a standardised routine for each researcher while collecting patient-data, e.g., aiming to avoid comments or discussions on the intervention (5A vs. ABC training) while talking to GPs or patients. Moreover, self-reflection and peer supervision during team meetings will be used to minimize and document potential biases on a regular basis.

In this study, primary and important secondary outcomes are measured immediately following the training. Any effect on these outcomes is therefore expected to decrease after a longer period of time. If this study shows that our training is effective in increasing the rates of delivery of brief GP advice on quitting, long-term effects need to be evaluated with further research.

Smoking status in this study, measured as a secondary outcome at follow-up, will be assessed by self-report and not be biochemically verified. However, over-reporting of smoking abstinence may be assumed to be equally distributed over the study arms, may not differ between pre- and post-training measurement, and in studies with no or only limited face-to-face contact, the use of biochemical verification is not necessarily required [47].

Finally, the study will be conducted in the federal state of NRW. Results can thus not be entirely generalised to GP practices across all provinces of Germany. However, NRW is the fourth largest of the German federal states and represents an agglomeration of urban and rural areas with a broad socioeconomic variability. Sound representativeness of data is therefore assumed.

If the training increases the rates of delivery of stop-smoking advice, ideally together with a recommendation of an evidence-based smoking cessation treatment, it would offer a low-threshold strategy for the implementation of the national clinical guideline on treating tobacco addition in primary care as well as of the implementation recommendations of the WHO-FCTC Article 14. Should one of the training methods, 5A or ABC, prove superior, a more specific guideline recommendation can be proposed for patient care. However, even a broad implementation of an effective intervention in primary care cannot entirely cover the lack of reimbursement of costs that come along with smoking cessation treatment and the absence of established specialist cessation services which have been shown to be highly effective [48]. This remains a debate for politics.

## Additional files

Additional file 1: BaselineSurvey_ABCII_German. (PDF 542 kb)

Additional file 2: BaselineSurvey_ABCII_Engl. (PDF 560 kb)

Additional file 3: Follow-upSurveyWeek4_ABCII_German. (PDF 486 kb)

Additional file 4: Follow-upSurveyWeek4_ABCII_Engl. (PDF 537 kb)

## Data Availability

Data sharing is not applicable to this article as this is a study protocol. Study materials such as the training manual, handouts, or questionnaires are available on reasonable request from the corresponding author. Also, it is intended to process the training manual at the end of the trial and to provide access to all medical teaching institutions in Germany, so that the training can be offered to GPs nationwide.

## Abbreviations

BCT: Behaviour Change Techniques
BMG: “Bundesministerium für Gesundheit” (German Federal Ministry of Health)
CME: Continuing Medical Education
CoMeD: Communication in Medical education Düsseldorf
CONSORT: Consolidated Standards of Reporting Trials
cRCT: Cluster Randomised Controlled Trial
DEBRA: “DEutsche Befragung zum Rauchverhalten” (German Study on Tobacco Use)
EQ-5D: EuroQol 5 Dimensions
FCTC: Framework Convention on Tobacco Control
GPs: General practitioner
HHU: Heinrich-Heine-University
HRQL: Health-related quality of life
ifam: “Institut für Allgemeinmedizin” (Institute of General Practice)
MTSS: Motivation to Stop Scale
NRT: Nicotine replacement therapy
SD: Standard deviation
SES: Socioeconomic status
SP: Standardised patient
SUTS: Strength of Urges to Smoke Scale
WHO: World Health Organisation
