# Supplementary material for: Effectiveness of training general practitioners to improve the implementation of brief stop-smoking advice in German primary care: study protocol of a pragmatic, 2-arm cluster randomised controlled trial (the ABCII trial)
Source: BMC Fam Pract. 2019 Jul 27;20:107. doi: 10.1186/s12875-019-0986-8 (PMC6660716; doi:10.1186/s12875-019-0986-8)
Supplement: Supplementary file 1 — BaselineSurvey_ABCII_German. (PDF 542 kb) [file 12875_2019_986_MOESM1_ESM.pdf]

This work is licensed under the Creative Commons Attribution
BY NC ND

Sehr geehrte Patientin, sehr geehrter Patient!

Ihre Hausärztin/Ihr Hausarzt nimmt an einer wissenschaftlichen Befragung des Instituts für Allgemeinmedizin des Universitätsklinikums Düsseldorf teil. Das **Thema der Befragung lautet „Arzt-Patienten-Gespräch zum Gesundheitsverhalten"**. Ziel unserer Forschung ist es, den Einfluss solcher Gespräche auf bestimmte Gesundheitsverhaltensweisen von Patienten/innen zu untersuchen.

Hierfür bitten wir Sie, einige Fragen zu Ihrem Gesundheitsverhalten sowie zu Ihrer Ausbildung und Ihrem Beruf zu beantworten. Ihre Teilnahme ist freiwillig und wird keinen Einfluss auf Ihre Behandlung haben. Es werden keine weiteren medizinischen Daten erhoben. Ihre Hausärztin/Ihr Hausarzt unterliegt weiterhin der Schweigepflicht. Auch werden keine Aussagen, die Sie über ihren Hausarzt/ihre Hausärztin machen, von uns weitergegeben. **Sie können Ihre Teilnahme jederzeit und ohne Angabe von Gründen widerrufen, ohne dass dies nachteilige Folgen für Sie hat**.

Sollten Sie mit der Teilnahme an der Befragung und der Einwilligungserklärung zum Datenschutz einverstanden sein, bitten wir Sie, dies mit Ihrer Unterschrift auf der zweiten Seite zu bestätigen. **Ihre Unterschrift bestätigt ebenfalls, dass wir Ihnen in 1, 3 und 6 Monaten nochmals je einen Kurzfragebogen (10minütige Dauer) mit der Post zusenden dürfen.** Um Ihnen die Rückantwort zu erleichtern, wird diesem Schreiben bereits ein frankierter und adressierter Rückumschlag beiliegen. Wenn Sie wünschen, informieren wir Sie am Ende der Studie (Studiendauer 3 Jahre) gerne über die Ergebnisse der Befragung.

Geburtsdatum: ____________________ **Geschlecht**: ☐ weiblich / ☐ männlich

Vor- und Nachname:

☒ ____

Anschrift (für die postalische Nachbefragung):

☒ ____

(Straße, Hausnummer, Postleitzahl, Stadt)

Heute behandelnde Hausärztin/behandelnder Hausarzt: ________________________________

Interviewer/Interviewerin: ____ ☐**Prä** ☐ **Post**

Kontakt für Rückfragen oder Widerruf: Dr. Sabrina Kastaun, Institut für Allgemeinmedizin (ifam),
Telefon: 0211/81 19527

Einwilligungserklärung zum Datenschutz

Mir ist bekannt, dass bei dieser Studie personenbezogene Daten über mich erhoben, gespeichert und ausgewertet werden sollen. Diese Daten umfassen ausschließlich die Angaben, die ich in dem heutigen Fragebogen und in den Nachbefragungen selbst mache. Die Verwendung der Angaben erfolgt nach gesetzlichen Bestimmungen und setzt vor der Teilnahme an der Studie folgende freiwillig abgegebene Einwilligungserklärung voraus. Dies heißt: **Ohne die nachfolgende Einwilligung kann ich nicht an der Studie teilnehmen.**

1) Ich erkläre mich damit einverstanden, dass im Rahmen dieser Studie erhobene Daten in Papierform und auf elektronischen Datenträgern vom Institut für Allgemeinmedizin der Heinrich-Heine-Universität Düsseldorf (Projektverantwortung: Prof. Dr. Daniel Kotz) aufgezeichnet und gespeichert werden. Die erhobenen Daten dürfen nur pseudonymisiert (ohne Namen, mit einem Code verschlüsselt) und nur für wissenschaftliche Zwecke (z.B. für die Veröffentlichung in Fachzeitschriften oder auf Konferenzen) verwendet werden. Die Zuordnung von meinem Pseudonym (Code) zu meinem Namen erfolgt in einer getrennten Datei, die nur Mitgliedern des Studienzentrums zugänglich ist.

2) Ich bin darüber aufgeklärt worden, dass ich meine Einwilligung in die Aufzeichnung, Speicherung und Verwendung meiner Daten jederzeit widerrufen kann. Bei einem Widerruf werden meine Daten unverzüglich gelöscht.

3) Ich bin damit einverstanden, dass meine Daten nach Beendigung oder Abbruch der Studie 10 Jahre aufbewahrt werden. Danach werden meine personenbezogenen Daten gelöscht, soweit dem nicht gesetzliche, satzungsgemäße oder vertragliche Aufbewahrungsfristen entgegenstehen.

Unterschrift ☒ ________________________________

Kontakt für Rückfragen oder Widerruf: Dr. Sabrina Kastaun, Institut für Allgemeinmedizin (ifam),
Telefon: 0211/81 19527

Kurzbefragung „Arzt-Patienten-Gespräch zum Gesundheitsverhalten“

1) Welchen **höchsten** allgemeinbildenden Schulabschluss haben Sie oder streben Sie zukünftig an? Machen Sie bitte **nur eine Angabe**.

☐ Keinen Schulabschluss
☐ Hauptschulabschluss (Volksschulabschluss, Polytechnische Oberschule mit Abschluss 8. oder 9. Klasse)
☐ Mittlere Reife, Realschulabschluss, Fachschulreife (Polytechnische Oberschule mit Abschluss 10. Klasse)
☐ Fachhochschulreife, Abschluss einer Fachoberschule
☐ Abitur (allgemeine oder fachgebundene Hochschulreife)

2) Welchen **höchsten** Berufs- oder Ausbildungsabschluss haben Sie oder streben Sie zukünftig an? Machen Sie bitte **nur eine Angabe**.

☐ Noch keinen, ich bin noch Schüler/-in (auch Berufsschule)
☐ Noch keinen, ich befinde mich noch in beruflicher Ausbildung (Berufsvorbereitungsjahr, Auszubildende/r, Student/-in, Lehrling, Praktikant/-in)
☐ Ich habe keinen beruflichen Abschluss und bin auch nicht in beruflicher Ausbildung/Lehre oder noch Schüler/-in
☐ Berufliche oder schulische Ausbildung bzw. Lehre abgeschlossen
☐ Ausbildung an einer Meister-, Technikerschule, Berufs- oder Fachakademie abgeschlossen
☐ Fachhochschulabschluss (an Fachhochschule, Fernstudium) (z.B. Diplom, Master)
☐ Hochschulabschluss an Universität (z.B. Bachelor, Master, Diplom, Magister, Staatsexamen)

3) Welche Erwerbssituation passt momentan für Sie? Bitte beachten Sie, dass unter Erwerbstätigkeit jede bezahlte bzw. mit einem Einkommen verbundene Tätigkeit verstanden wird. Machen Sie bitte **nur eine Angabe**.

☐ Vollzeiterwerbstätig
☐ Teilzeiterwerbstätig (auch Altersteilzeit etc.)
☐ Noch in beruflicher Ausbildung (Lehre, Volontariat, Praktisches Jahr oder ähnliches)
☐ Geringfügig erwerbstätig (Gelegenheitsbeschäftigung, Mini-Job, 400-Euro-Job, Ein-Euro-Job)
☐ Mutterschafts- oder Vaterschaftsurlaub, Erziehungsurlaub oder sonstige Beurlaubung
☐ Nicht erwerbstätig, sondern
a) ☐ Hausfrau/Hausmann
b) ☐ Schüler/-in, Student/-in, berufliche Umschulung, Praktikant/-in, Wehrdienst/ Zivildienst, FSJ
c) ☐ Arbeitssuchend oder Arbeitslos (Arbeitslosengeld I oder II „Harz 4“)
d) ☐ Rentner/Pensionär/-in, Vorruhestand (ohne Nebenverdienst)
e) ☐ Dauerhaft erwerbsunfähig

Bitte weiter auf nächster Seite!

4.) Rauchen Sie Tabak (z.B. Zigaretten, Zigarre, Pfeife)? Machen Sie bitte **nur eine Angabe**.

☐ Ja, ich rauche derzeit täglich
☐ Ja, ich rauche derzeit gelegentlich
☐ Nein, aber ich habe früher täglich oder gelegentlich geraucht → **für Sie endet der Fragebogen hier**
☐ Nein, ich habe noch nie täglich oder gelegentlich geraucht → **für Sie endet der Fragebogen hier**

5.) Wie lang ist es her, dass Sie zum letzten Mal eine Zigarette oder eine andere Form von Tabak geraucht haben? Machen Sie bitte **nur eine Angabe.**

☐ Weniger als 24 Stunden
☐ Zwischen einem Tag und einer Woche
☐ Zwischen einer Woche und vier Wochen → **für Sie endet der Fragebogen hier**
☐ Zwischen vier Wochen und 12 Wochen → **für Sie endet der Fragebogen hier**
☐ Zwischen 12 Wochen und sechs Monaten → **für Sie endet der Fragebogen hier**
☐ Länger als sechs Monate → **für Sie endet der Fragebogen hier**

6.) Wie viele Filterzigaretten oder selbstgedrehte oder selbstgestopfte Zigaretten oder Zigarren/ Pfeifen rauchen Sie normalerweise oder rauchten Sie bevor Sie kürzlich mit dem Rauchen aufhörten? Geben Sie die Menge pro Tag oder, wenn Sie gelegentlicher Raucher sind, pro Woche oder Monat an. **Mehrere Antworten möglich.**

Filterzigaretten:	pro Tag _____	oder pro Woche _____	oder pro Monat ______
Selbst gedrehte Zigaretten:	pro Tag _____	oder pro Woche _____	oder pro Monat ______
Selbst gestopfte Zigaretten:	pro Tag _____	oder pro Woche _____	oder pro Monat ______
Zigarren:	pro Tag _____	oder pro Woche _____	oder pro Monat ______
Zigarillos:	pro Tag _____	oder pro Woche _____	oder pro Monat ______
Pfeife:	pro Tag _____	oder pro Woche _____	oder pro Monat ______
Shishas:	pro Tag _____	oder pro Woche _____	oder pro Monat ______

7.) Wie häufig haben Sie in den vergangenen 24 Stunden das Verlangen verspürt zu rauchen? Machen Sie bitte **nur eine Angabe.**

☐ Überhaupt nicht → **weiter mit Frage 9**
☐ Selten
☐ Manchmal
☐ Häufig
☐ Fast immer
☐ Immer

Bitte weiter auf nächster Seite!

8.) Wie stark war dieses Verlangen im Allgemeinen? Machen Sie bitte **nur eine Angabe.**

☐ Leicht
☐ Mittelstark
☐ Stark
☐ Sehr stark
☐ Extrem stark

9.) Welche der folgenden Aussagen trifft auf Sie zu? Machen Sie bitte **nur eine Angabe.**

☐ Ich will nicht mit dem Rauchen aufhören.
☐ Ich sollte mit dem Rauchen aufhören, aber ich möchte eigentlich nicht.
☐ Ich will mit dem Rauchen aufhören, habe aber noch nicht darüber nachgedacht, wann.
☐ Ich will **unbedingt** mit dem Rauchen aufhören, habe aber noch nicht darüber nachgedacht, wann.
☐ Ich will mit dem Rauchen aufhören und hoffe, dies in naher Zukunft zu tun.
☐ Ich will **unbedingt** mit dem Rauchen aufhören und habe vor, dies in den nächsten drei Monaten zu tun.
☐ Ich will **unbedingt** mit dem Rauchen aufhören und habe vor, dies im nächsten Monat zu tun.

10.) Hat Ihre Hausärztin/ Ihr Hausarzt heute während der Sprechstunde mit Ihnen über Ihr Rauchverhalten gesprochen? Machen Sie bitte **nur eine Angabe.**

☐ Ja, meine Hausärztin / mein Hausarzt hat mich auf mein Rauchverhalten angesprochen → **weiter mit Frage 11**
☐ Ja, ich habe meine Hausärztin / mein Hausarzt auf mein Rauchverhalten angesprochen → **weiter mit Frage 11**
☐ Nein, meine Hausärztin / mein Hausarzt und ich haben nicht über mein Rauchverhalten gesprochen → **weiter mit Frage 14**
☐ Ich kann mich nicht mehr daran erinnern → **weiter mit Frage 14**

11.) Wenn Ihre Hausärztin/ Ihr Hausarzt mit Ihnen über Ihr Rauchverhalten gesprochen hat, welche der folgenden Situationen trifft auf dieses Gespräch zu?
Beantworten Sie bitte jede Frage.

a. Hat Ihre Hausärztin/ Ihr Hausarzt Sie danach gefragt, ob Sie rauchen?
☐ Ja
☐ Nein
☐ Ich kann mich nicht mehr daran erinnern

Bitte weiter auf nächster Seite!

b. Hat Ihre Hausärztin/ Ihr Hausarzt Ihnen geraten mit dem Rauchen aufzuhören?

- ☐ Ja
- ☐ Nein
- ☐ Ich kann mich nicht mehr daran erinnern

c. Hat Ihre Hausärztin/ Ihr Hausarzt Sie gefragt, ob Sie mit dem Rauchen aufhören wollen?

- ☐ Ja
- ☐ Nein
- ☐ Ich kann mich nicht mehr daran erinnern

d. Hat Ihre Hausärztin/ Ihr Hausarzt mit Ihnen weitere Termine verabredet, um das Thema Rauchen oder einen Rauchstopp noch einmal mit Ihnen zu besprechen?

- ☐ Ja
- ☐ Nein
- ☐ Ich kann mich nicht mehr daran erinnern

12.) Hat Ihre Hausärztin/ Ihr Hausarzt Ihnen Unterstützung oder Behandlung beim Aufhören angeboten? Machen Sie bitte **nur eine Angabe.**

- ☐ Ja, meine Hausärztin / mein Hausarzt hat mir Nikotinpflaster oder -kaugummis empfohlen.
- ☐ Ja, meine Hausärztin / mein Hausarzt hat mir ein Medikament (Vareniclin/Champix oder Bupropion/Zyban) empfohlen oder verschrieben.
- ☐ Ja, meine Hausärztin / mein Hausarzt hat mir eine weiterführende Rauchstopp-Beratung oder Behandlung in der eigenen Praxis empfohlen (zum Beispiel Einzel- oder Gruppentherapie).
- ☐ Ja, meine Hausärztin / mein Hausarzt hat mir eine weiterführende Rauchstopp-Beratung oder Behandlung außerhalb der Praxis empfohlen (zum Beispiel Einzel- oder Gruppentherapie).
- ☐ Ja, meine Hausärztin / mein Hausarzt hat mir Nikotinpflaster oder -kaugummis oder ein Medikament und eine weiterführende Rauchstopp-Beratung oder Behandlung (in der eigenen Praxis oder außerhalb der Praxis) empfohlen.
- ☐ Nein, meine Hausärztin / mein Hausarzt hat mir keine Behandlung angeboten.
- ☐ Ja, meine Hausärztin / mein Hausarzt hat mir eine Infobroschüre/ Flyer zum Thema Rauchentwöhnung mitgegeben.
- ☐ Ich kann mich nicht mehr daran erinnern.

13.) Auf einer Schulnotenskala (1 = sehr zufrieden, 6 = überhaupt nicht zufrieden), wie zufrieden sind Sie mit diesem Gespräch über ihr Rauchverhalten gewesen?

Note: ________

Bitte weiter auf nächster Seite!

Die folgenden Aussagen beziehen sich abschließend auf ihren heutigen, allgemeinen Gesundheitszustand

14.) Bitte kreuzen Sie unter jeder Überschrift DAS Kästchen an, das Ihre Gesundheit **HEUTE** am besten beschreibt? Machen Sie bitte **nur eine Angabe pro Überschrift A) bis E).**

A) BEWEGLICHKEIT / MOBILITÄT

☐ Ich habe keine Probleme herumzugehen

☐ Ich habe leichte Probleme herumzugehen

☐ Ich habe mäßige Probleme herumzugehen

☐ Ich habe große Probleme herumzugehen

☐ Ich bin nicht in der Lage herumzugehen

B) FÜR SICH SELBST SORGEN

☐ Ich habe keine Probleme, mich selbst zu waschen oder anzuziehen

☐ Ich habe leichte Probleme, mich selbst zu waschen oder anzuziehen

☐ Ich habe mäßige Probleme, mich selbst zu waschen oder anzuziehen

☐ Ich habe große Probleme, mich selbst zu waschen oder anzuziehen

☐ Ich bin nicht in der Lage, mich selbst zu waschen oder anzuziehen

C) ALLTÄGLICHE TÄTIGKEITEN (z.B. Arbeit, Studium, Hausarbeit, Familien- oder Freizeitaktivitäten)

☐ Ich habe keine Probleme, meinen alltäglichen Tätigkeiten nachzugehen

☐ Ich habe leichte Probleme, meinen alltäglichen Tätigkeiten nachzugehen

☐ Ich habe mäßige Probleme, meinen alltäglichen Tätigkeiten nachzugehen

☐ Ich habe große Probleme, meinen alltäglichen Tätigkeiten nachzugehen

☐ Ich bin nicht in der Lage, meinen alltäglichen Tätigkeiten nachzugehen

D) SCHMERZEN / KÖRPERLICHE BESCHWERDEN

☐ Ich habe keine Schmerzen oder Beschwerden

☐ Ich habe leichte Schmerzen oder Beschwerden

☐ Ich habe mäßige Schmerzen oder Beschwerden

☐ Ich habe starke Schmerzen oder Beschwerden

☐ Ich habe extreme Schmerzen oder Beschwerden

Bitte weiter auf nächster Seite!

E) ANGST / NIEDERGESCHLAGENHEIT

☐ Ich bin nicht ängstlich oder deprimiert

☐ Ich bin ein wenig ängstlich oder deprimiert

☐ Ich bin mäßig ängstlich oder deprimiert

☐ Ich bin sehr ängstlich oder deprimiert

☐ Ich bin extrem ängstlich oder deprimiert

15.) Wir möchten herausfinden, wie gut oder schlecht Ihre Gesundheit **HEUTE** ist?

- Stellen Sie sich eine Skala vor (wie ein stehendes Lineal)
- Am oberen Ende steht die 100, am unteren Ende sehen Sie die Null (0)
- 100 (oben) ist die <u>beste</u> Gesundheit, die Sie sich vorstellen können.
  0 (unten) ist die <u>schlechteste</u> Gesundheit, die Sie sich vorstellen können.
- Welche Zahl auf der Skala zwischen 0 und 100 spiegelt Ihre heutige Gesundheit wider?

IHRE GESUNDHEIT HEUTE = ☐

Beste Gesundheit, die Sie sich vorstellen können

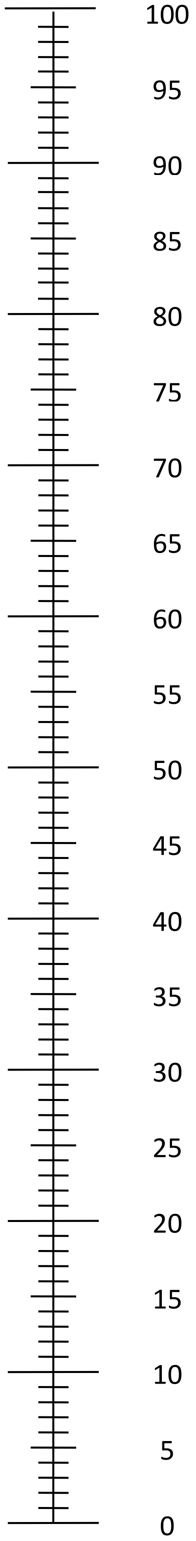

Schlechteste Gesundheit, die Sie sich vorstellen können

Nachträgliche Aufklärung der Studienteilnehmer
über den vollständigen Inhalt der Befragung

Haben Sie vielen Dank für die Zeit, die Sie sich heute genommen haben, um uns bei unserer Studie zu unterstützen.

In der Aufklärung zu der Studie haben Sie erfahren, dass wir eine Studie durchführen zum Thema „Arzt-Patienten-Gespräch zum Gesundheitsverhalten“.

Das klingt etwas „schwammig“ und Sie werden sicherlich gemerkt haben, dass wir Sie heute gezielt nur nach dem Rauchen gefragt haben. Genauer gesagt, geht es in unserer Studie nämlich darum, dass wir Hausärzte/innen darin schulen, wie sie ihren rauchenden Patienten/innen während der Sprechstunde eine Kurzberatung zum Rauchstopp anbieten können. Dabei werden wir alle Hausärzte/innen, die an der Studie teilnehmen, in zwei Gruppen aufteilen und sie auf unterschiedliche Arten darin schulen, solch eine Beratung anzubieten.

Für uns ist es wichtig herauszufinden, ob und wie häufig die Hausärzte/innen vor bzw. nach einer Schulung ihren rauchenden Patienten/innen solch eine Rauchstoppberatung anbieten, auf welche Weise sie das tun, und wie Patienten/innen dann solch eine Beratung annehmen. Dafür befragen wir Sie als Patientin/Patient mehrfach, nachdem Sie bei ihrer Hausärztin/ ihrem Hausarzt in der Sprechstunde waren.

Haben Sie dazu noch Fragen oder war das soweit verständlich? Ich weise Sie nochmals darauf hin, dass ihre Teilnahme an der Befragung freiwillig ist und ihre Hausärztin/ihr Hausarzt weiterhin der Schweigepflicht unterliegt.
