# Supplementary material for: Effectiveness of training general practitioners to improve the implementation of brief stop-smoking advice in German primary care: study protocol of a pragmatic, 2-arm cluster randomised controlled trial (the ABCII trial)
Source: BMC Fam Pract. 2019 Jul 27;20:107. doi: 10.1186/s12875-019-0986-8 (PMC6660716; doi:10.1186/s12875-019-0986-8)
Supplement: Supplementary file 2 — BaselineSurvey_ABCII_Engl. (PDF 560 kb) [file 12875_2019_986_MOESM2_ESM.pdf]

Questionnaire (Translation from German)

This work is licensed under the Creative Commons Attribution
BY NC ND

Physician-patient communication on health behaviour

1) What is the **highest** level of school education you received so far, <u>or</u> you are aiming at in the future? Please choose <u>**only one answer.**</u>

☐ No qualification
☐ Junior high school equivalent
☐ Secondary school equivalent
☐ Advanced technical college equivalent
☐ High school equivalent

2) What is the **highest** job or professional education level you received so far, <u>or</u> you are aiming for in the future? Please choose <u>**only one answer.**</u>

☐ No qualification yet, I am still in school (also vocational college or trade school)
☐ No qualification yet, I am currently participating in professional training (apprentice, trainee, student, intern)
☐ I do <u>not</u> have a training qualification and I am <u>not</u> participating in professional training as an apprentice, trainee, or pupil
☐ Professional or academic training degree
☐ Master-, technician- or vocational school degree
☐ Polytechnic degree / University of applied science (e.g., Diploma, Master)
☐ University degree (e.g., Bachelor, Master, state examination)

3) Which employment situation currently applies to you? Please note, that employed means any payed occupation or XXX. Please choose <u>**only one answer.**</u>

☐ Full time occupation
☐ Part time occupation (also partial retirement or comparable)
☐ Currently participating in a professional training (professional training, voluntary service, practical year or comparable)
☐ Minimal employment (occasional job, mini-job, 450-Euro-based-job, one-Euro-job)
☐ Maternity- or paternity leave, childcare leave or leave of absence for other reasons
☐ Not employed, but
  a) ☐ Housewife / homemaker
  b) ☐ Pupil, student, professional retraining, intern, military service/civil service, social year
  c) ☐ Seeking work or unemployed ("Arbeitslosengeld I or II", "Harz 4")
  d) ☐ Retiree, early retiree (without other income)
  e) ☐ Permanent occupational disability

Please continue to the next page!

4) Do you smoke tobacco (e.g., cigarettes, cigars, pipe)? Please choose **only one answer.**

☐ Yes, I currently smoke every day
☐ Yes, I currently smoke occasionally
☐ No, currently not, but I used to smoke daily or occasionally → **the questionnaire ends here**
☐ No, I have never smoked daily or occasionally → **the questionnaire ends here**

5) How long has it been, since you last smoked a cigarette or any other tobacco product? Please choose **only one answer.**

☐ Less than 24 hours
☐ Between one day and one week
☐ Between one week and four weeks → **the questionnaire ends here**
☐ Between four weeks and 12 weeks → **the questionnaire ends here**
☐ Between 12 weeks and six months → **the questionnaire ends here**
☐ Longer than six months ago → **the questionnaire ends here**

6) How many filter cigarettes or self-rolled or stuffed cigarettes or cigars / pipes do you usually smoke, or did you smoke before you quit recently? Please enter the quantity per day or, in case you are an occasional smoker, per week or per month. **Multiple answers allowed.**

Filter cigarettes:	per day _____	or per week _____	or per month ______
Self-rolled cigarettes:	per day _____	or per week _____	or per month ______
Self-stuffed cigarettes:	per day _____	or per week _____	or per month ______
Cigars:	per day _____	or per week _____	or per month ______
Pipes:	per day _____	or per week _____	or per month ______
Shishas:	per day _____	or per week _____	or per month ______
Cigarillos:	per day _____	or per week _____	or per month ______

7) How much of the time have you felt the urge to smoke in the past 24 hours? Please choose **only one answer.**

☐ Not at all → **continue with question 9**
☐ A little of the time
☐ Some of the time
☐ A lot of the time
☐ Almost of the time
☐ All the time

Please continue to the next page!

8) In general, how strong have the urges to smoke been? Please choose **<u>only one answer.</u>**

☐ Slight
☐ Moderate
☐ Strong
☐ Very strong
☐ Extremely strong

9) Which of the following situations best describes you? Please choose **<u>only one answer.</u>**

☐ I don't want to stop smoking.
☐ I think I should stop smoking but I don't really want to.
☐ I want to stop smoking but haven't thought about when.
☐ I **really** want to stop smoking, but I don't know when I will.
☐ I do want to stop smoking, and I hope to do so soon.
☐ I **really** want to stop smoking and intend to do so within the next three months.
☐ I **really** want to Stop smoking and intend to do so within the next month.

10) Did your general practitioner / family doctor talk to you about your smoking behaviour during your consultation today? Please choose **<u>only one answer.</u>**

☐ Yes, <u>my general practitioner addressed</u> my smoking behaviour → **continue with question 11**
☐ Yes, <u>I addressed</u> my general practitioner regarding my smoking behaviour → **continue with question 11**
☐ No, my general practitioner and I did <u>not</u> talk about my smoking behaviour → **continue with question 14**
☐ I cannot remember → **continue with question 14**

11) <u>If your general practitioner addressed your smoking behaviour,</u> which of the following situations applies for this conversation? **<u>Please answer each question.</u>**

a. Did your general practitioner asked you whether you smoke?
☐ Yes
☐ No
☐ I cannot remember

Please continue to the next page!

b. Did your general practitioner advise you to quit smoking?

☐ Yes
☐ No
☐ I cannot remember

c. Did your general practitioner ask you, if you want to quit smoking?

☐ Yes
☐ No
☐ I cannot remember

d. Did your general practitioner schedule further appointments, to discuss the topic smoking or quitting with you?

☐ Yes
☐ No
☐ I cannot remember

12) Did your general practitioner offer stop-smoking support or treatment to you? Please choose **only one answer.**

☐ Yes, my general practitioner recommended nicotine patches or gums.
☐ Yes, my general practitioner recommended or prescribed medication (Vareniclin/Champix or Bupropion/Zyban).
☐ Yes, my general practitioner recommended further smoking cessation counselling or treatment in her/his own practice (e.g., single or group therapy).
☐ Yes, my general practitioner recommended further smoking cessation counselling or treatment outside her/his practice (e.g., single or group therapy).
☐ Yes, my general practitioner recommended nicotine patches, gums, or medication and further smoking cessation counselling or treatment (in or outside her/his own practice).
☐ No, my general practitioner did not offer any treatment.
☐ Yes, my general practitioner gave me a brochure/leaflet with information on smoking cessation.
☐ I cannot remember.

13) On a school-grade scale (1 = totally satisfied, 6 = not at all satisfied), how satisfied are you with this conversation about your smoking behaviour? Please choose **only one answer.**

Grade: _________

Please continue to the next page!

The following final set of questions is related to your current <u>general state of health</u>*

14) Under each heading, please tick the ONE box that describes your health **TODAY** best. Please choose **<u>only one answer</u>** per heading **<u>A) to E).</u>**

A) MOBILITY / FLEXIBILITY

☐ I have no problems in walking around

☐ I have slight problems in walking around

☐ I have moderate problems in walking around

☐ I have severe problems in walking around

☐ I am unable to walk around

B) SELF-CARE

☐ I have no problems washing or dressing myself

☐ I have slight problems washing or dressing myself

☐ I have moderate problems washing or dressing myself

☐ I have severe problems washing or dressing myself

☐ I am unable to wash or dress myself

C) USUAL ACTIVITIES (e.g., work, study, housework, family or leisure activities)

☐ I have no problems doing my usual activities

☐ I have slight problems doing my usual activities

☐ I have moderate problems doing my usual activities

☐ I have severe problems doing my usual activities

☐ I am unable to do my usual activities

D) PAIN / DISCOMFORT

☐ I have no pain or discomfort

☐ I have slight pain or discomfort

☐ I have moderate pain or discomfort

☐ I have severe pain or discomfort

☐ I have extreme pain or discomfort

Please continue to the next page!

E) ANXIETY / DEPRESSION

☐ I am not anxious or depressed

☐ I am slightly anxious or depressed

☐ I am moderately anxious or depressed

☐ I am very anxious or depressed

☐ I am extremely anxious or depressed

15) We would like to know how good or poor your health **TODAY**.

- Imagine a scale (like a standing up ruler)
- The top of the scale is marked with 100, the bottom with zero (0)
- 100 (top) means the best health you can imagine.
  0 (bottom) means the worst health you can imagine.
- Mark an X on the scale to indicate how is your health today
- Now, please note the number your marked in the box below.

YOUR HEALTH TODAY = ☐

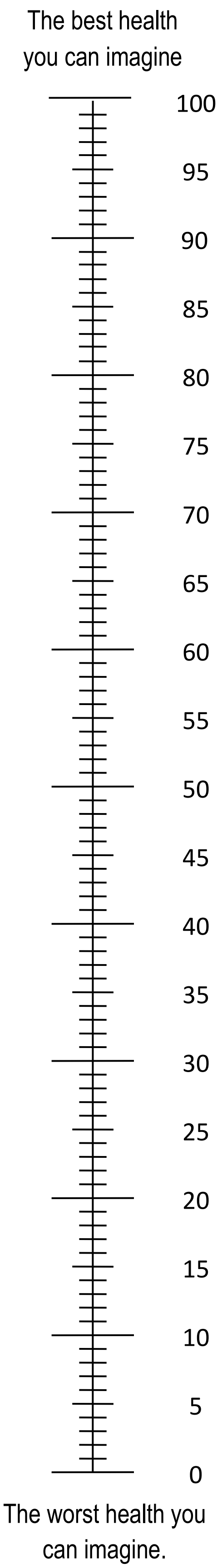

*Rabin R, de Charro F. EQ-5D: a measure of health status from the EuroQol Group. Ann Med 2001;33:337-43.
