# Supplementary material for: Effectiveness of training general practitioners to improve the implementation of brief stop-smoking advice in German primary care: study protocol of a pragmatic, 2-arm cluster randomised controlled trial (the ABCII trial)
Source: BMC Fam Pract. 2019 Jul 27;20:107. doi: 10.1186/s12875-019-0986-8 (PMC6660716; doi:10.1186/s12875-019-0986-8)
Supplement: Supplementary file 3 — Follow-upSurveyWeek4_ABCII_German. (PDF 486 kb) [file 12875_2019_986_MOESM3_ESM.pdf]

UKD Universitätsklinikum Düsseldorf
HEINRICH HEINE UNIVERSITÄT DÜSSELDORF
ifam INSTITUT FÜR ALLGEMEINMEDIZIN

Arzt-Patienten-Gespräch zum Gesundheitsverhalten

(Nachbefragung 4. Woche)

This work is licensed under the Creative Commons Attribution
CC BY NC ND

Datum von heute:__________________________ **Pat. ID (vom Institut zu ergänzen):** _______________

Geburtsdatum:____________________________

Geschlecht: ☐ weiblich / ☐ männlich

(Bitte gehen Sie auf den folgenden Seiten die Fragen einzeln durch und kreuzen Sie die Antworten an, die für Sie zutreffend sind)

1.) Versuchen Sie sich bitte an Ihren Besuch bei Ihrer Hausärztin/ Ihrem Hausarzt, **vor ca. 4 Wochen**, zu erinnern. Dort haben Sie freundlicherweise an einer kurzen Befragung teilgenommen, die von einem/r Mitarbeiter/in vom Institut für Allgemeinmedizin (ifam) durchgeführt wurde.

Haben Sie **seit diesem Termin vor ca. 4 Wochen** einen oder mehrere Versuche unternommen mit dem Tabakrauchen aufzuhören? Machen Sie bitte **nur eine Angabe.**

☐ Ja
☐ Nein → **weiter mit Frage 9**

Die nachfolgenden Fragen 2-8 bitte nur beantworten, wenn Sie seit dem Termin bei Ihrer Hausärztin/ Ihrem Hausarzt vor ca. 4 Wochen mindestens einmal versucht haben, mit dem Rauchen aufzuhören (also Frage 1 mit „Ja" beantwortet haben).

2.) Können Sie angeben, wann Sie (nach diesem Arzttermin vor ca. 4 Wochen) den letzten Rauchstopp-Versuch gestartet haben? Sollten Sie dies nicht genau wissen, probieren sie eine ungefähre Angabe zu machen. Machen Sie bitte **nur eine Angabe.**

vor _______ Tagen **oder** vor _______ Wochen

3.) Können Sie angeben, was der **Hauptgrund** für Sie war diesen letzten Rauchstopp-Versuch zu unternehmen? Machen Sie bitte **nur eine Angabe.**

☐ Der Rat meiner Hausärztin/ meines Hausarztes mit dem Rauchen aufzuhören war für mich der Hauptgrund
☐ Das Rauchen wurde mir zu teuer
☐ Das Rauchen ist schädlich für meine Gesundheit
☐ Jemand aus meinem Familien- oder Bekanntenkreis hat mit dem Rauchen aufgehört
☐ Die Gesundheitswarnungen auf den Zigarettenschachteln bzw. auf den Tabakpäckchen
☐ Jemand aus meinem Familien- oder Bekanntenkreis ist an einer Krankheit erkrankt, die durch das Rauchen entstehen kann (z.B. Krebs)
☐ Die Tatsache, dass das Rauchen an immer mehr Orten verboten ist
☐ Ein anderer Hauptgrund

4.) Wie lange hat dieser letzte Versuch, mit dem Rauchen aufzuhören, angehalten? Machen Sie bitte **nur eine Angabe.**

☐ Ich bin immer noch rauchfrei
☐ Weniger als einen Tag
☐ Weniger als eine Woche
☐ Weniger als einen Monat

5.) Haben Sie seit unserer Befragung vor ca. 4 Wochen noch einmal Ihre Hausärztin/ Ihren Hausarzt aufgesucht (hauptsächlich oder nur, um sich beim Rauchstopp beraten zu lassen? Machen Sie bitte **nur eine Angabe.**

☐ Ja, und zwar ____ Mal (bitte nur die Anzahl der Besuche bei der Hausärztin/ dem Hausarzt angeben, die Sie hauptsächlich oder nur wegen Ihres Rauchstopp-Versuches seit unserer letzten Befragung gemacht haben)
☐ Nein

UKD Universitätsklinikum Düsseldorf HEINRICH HEINE UNIVERSITÄT DÜSSELDORF ifam INSTITUT FÜR ALLGEMEINMEDIZIN

Bitte senden Sie den ausgefüllten Fragebogen

in dem dafür vorgesehenen Rückumschlag

so bald wie Ihnen möglich an uns zurück.

Vielen Dank

10.) Wir möchten herausfinden, wie gut oder schlecht Ihre Gesundheit **HEUTE** ist?

- Diese Skala ist mit Zahlen von 0 bis 100 versehen.
- 100 ist die <u>beste</u> Gesundheit, die Sie sich vorstellen können.
  0 (Null) ist die <u>schlechteste</u> Gesundheit, die Sie sich vorstellen können.
- Bitte kreuzen Sie den Punkt auf der Skala an, der Ihre Gesundheit HEUTE am besten beschreibt.
- Jetzt tragen Sie bitte die Zahl, die Sie auf der Skala angekreuzt haben, in das Kästchen unten ein.

IHRE GESUNDHEIT HEUTE = ☐

Beste Gesundheit, die Sie sich vorstellen können

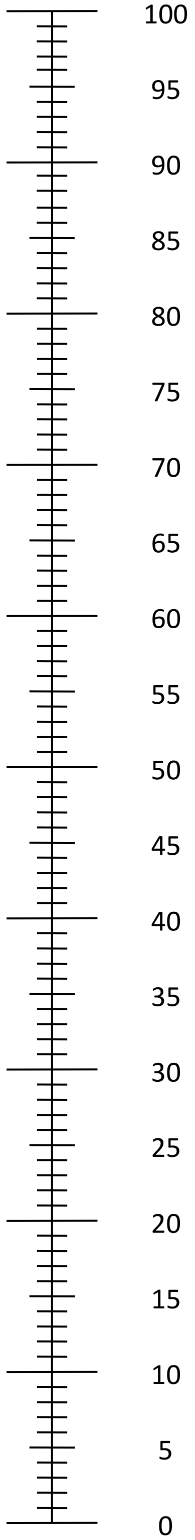

Schlechteste Gesundheit, die Sie sich vorstellen können

Germany (German) © 2009 EuroQol Group EQ-5D™ is a trade mark of the EuroQol Group

6.) Welche der folgenden Sachen/Methoden haben Sie bei diesem <u>letzten</u> Versuch unternommen, um Ihren Rauchstopp zu unterstützen? **<u>Mehrere Antworten möglich.</u>**

☐ Nikotinersatztherapie (z.B. Nikotinpflaster oder Nikotinkaugummis)
☐ Das Medikament Zyban (Bupropion)
☐ Das Medikament Champix (Vareniclin)
☐ Verhaltenstherapeutische Behandlung zur Rauchentwöhnung (z.B. Einzel- oder Gruppentherapie).
☐ Telefonische Beratung zur Rauchentwöhnung
☐ E-Zigarette mit Nikotin
☐ E- Zigarette ohne Nikotin
☐ Ein Buch zur Rauchentwöhnung (z.B. „Endlich Nichtraucher" von Allen Carr)
☐ Eine App oder ein Internetprogramm zur Rauchentwöhnung (auf einem Smartphone, Tablet oder PC)
☐ Akupunktur
☐ Hypnotherapie
☐ Heilpraktiker/in
☐ Keine der genannten Sachen/Methoden → **weiter mit Frage 9**

7.) Können Sie sich daran erinnern, wieviel Geld Sie für diese unterstützenden Sachen/Methoden bei diesem <u>letzten</u> Rauchstopp-Versuch <u>insgesamt</u> ausgegeben haben? **<u>Mehrere Antworten möglich.</u>**

______ € für Nikotinersatztherapie (z.B. Nikotinpflaster, Nikotinkaugummis, Nikotinsprays)

______ € für Medikamente (z.B. Zyban, Champix)

______ € für E-Zigaretten (sofern Sie als Hilfe bei der Rauchentwöhnung eingesetzt werden)

______ € für therapeutische Behandlungen (z.B. Einzel- oder Gruppentherapie)

______ € für andere Behandlungen (z.B. Akupunktur, Hypnotherapie, Heilpraktiker/in)

______ € für andere Sachen/Methoden (z.B. Buch zur Rauchentwöhnung oder kostenpflichtige App)

☐ Ich habe <u>kein</u> Geld ausgegeben → **weiter mit Frage 9**

☐ Ich kann mich nicht mehr erinnern → **weiter mit Frage 9**

Bitte weiter auf nächster Seite!

8.) Wenn Sie für eine oder mehrere dieser unterstützenden Sachen/Methoden bei diesem letzten Rauchstopp-Versuch Geld ausgegeben haben, hat Ihre Krankenkasse Ihnen davon etwas erstattet?

☐ Ja, und zwar insgesamt ________€

☐ Nein, ich habe alles aus der eigenen Tasche bezahlt

☐ Ich weiß nicht, ob meine Krankenkasse das erstattet

☐ Ich habe keinen Antrag zur Kostenerstattung bei meiner Krankenkasse eingereicht

☐ Ich kann mich nicht mehr erinnern

Die folgenden Aussagen beziehen sich abschließend auf ihren heutigen, allgemeinen Gesundheitszustand

9.) Bitte kreuzen Sie unter jeder Überschrift DAS Kästchen an, das Ihre Gesundheit **HEUTE** am besten beschreibt? Machen Sie bitte **nur eine Angabe pro Überschrift A) bis E).**

A) BEWEGLICHKEIT / MOBILITÄT

☐ Ich habe keine Probleme herumzugehen
☐ Ich habe leichte Probleme herumzugehen
☐ Ich habe mäßige Probleme herumzugehen
☐ Ich habe große Probleme herumzugehen
☐ Ich bin nicht in der Lage herumzugehen

B) FÜR SICH SELBST SORGEN

☐ Ich habe keine Probleme, mich selbst zu waschen oder anzuziehen
☐ Ich habe leichte Probleme, mich selbst zu waschen oder anzuziehen
☐ Ich habe mäßige Probleme, mich selbst zu waschen oder anzuziehen
☐ Ich habe große Probleme, mich selbst zu waschen oder anzuziehen
☐ Ich bin nicht in der Lage, mich selbst zu waschen oder anzuziehen

Germany (German) © 2009 EuroQol Group EQ-5D™ is a trade mark of the EuroQol Group

UKD Universitätsklinikum Düsseldorf
HEINRICH HEINE UNIVERSITÄT DÜSSELDORF
ifam INSTITUT FÜR ALLGEMEINMEDIZIN

C) ALLTÄGLICHE TÄTIGKEITEN (z.B. Arbeit, Studium, Hausarbeit, Familien- oder Freizeitaktivitäten)

☐ Ich habe keine Probleme, meinen alltäglichen Tätigkeiten nachzugehen
☐ Ich habe leichte Probleme, meinen alltäglichen Tätigkeiten nachzugehen
☐ Ich habe mäßige Probleme, meinen alltäglichen Tätigkeiten nachzugehen
☐ Ich habe große Probleme, meinen alltäglichen Tätigkeiten nachzugehen
☐ Ich bin nicht in der Lage, meinen alltäglichen Tätigkeiten nachzugehen

D) SCHMERZEN / KÖRPERLICHE BESCHWERDEN

☐ Ich habe keine Schmerzen oder Beschwerden
☐ Ich habe leichte Schmerzen oder Beschwerden
☐ Ich habe mäßige Schmerzen oder Beschwerden
☐ Ich habe starke Schmerzen oder Beschwerden
☐ Ich habe extreme Schmerzen oder Beschwerden

E) ANGST / NIEDERGESCHLAGENHEIT

☐ Ich bin nicht ängstlich oder deprimiert
☐ Ich bin ein wenig ängstlich oder deprimiert
☐ Ich bin mäßig ängstlich oder deprimiert
☐ Ich bin sehr ängstlich oder deprimiert
☐ Ich bin extrem ängstlich oder deprimiert

Germany (German) © 2009 EuroQol Group EQ-5D™ is a trade mark of the EuroQol Group

Bitte weiter auf nächster Seite!
