# Supplementary material for: Effectiveness of training general practitioners to improve the implementation of brief stop-smoking advice in German primary care: study protocol of a pragmatic, 2-arm cluster randomised controlled trial (the ABCII trial)
Source: BMC Fam Pract. 2019 Jul 27;20:107. doi: 10.1186/s12875-019-0986-8 (PMC6660716; doi:10.1186/s12875-019-0986-8)
Supplement: Supplementary file 4 — Follow-upSurveyWeek4_ABCII_Engl. (PDF 537 kb) [file 12875_2019_986_MOESM4_ESM.pdf]

Follow-up questionnaire week 4 (Translation from German)

This work is licensed under the Creative Commons Attribution
CC BY NC ND

Physician-patient communication on health behaviour

(Please answer the questions on the following pages point-by-point
and tick off the answers that apply to you)

1.) Please try to recall your last consultation with your general practitioner / family doctor, **about 4 weeks ago**. You kindly participated in a short survey, which was conducted by an employee of the Institute of General Practice (ifam).

Have you made one or more attempt(s) to quit smoking **since this last consultation 4 weeks ago**? Please choose **<u>only one answer</u>**.

☐ Yes
☐ No → **continue with question 9**

Please <u>only answer the following questions 2-8</u> if you have made at least one attempt to quit smoking since your last appointment with your general practitioner <u>about 4 weeks ago</u> (= if you answered question 1 with "Yes").

2.) Can you specify when (since this last consultation with your general practitioner about 4 weeks ago) you have started your <u>last</u> quit attempt? In case you cannot recall, please try to approximate. Please choose **<u>only one answer</u>**.

_______ days ago **or** _______ weeks ago

3.) Can you recall what the **main reason** for this <u>last</u> quit attempt was? Please choose **<u>only one answer</u>**.

☐ My general practitioner's advice to quit was the main reason
☐ Smoking became too expensive for me
☐ Smoking is harmful to my health
☐ Someone from my family or circle of acquaintances has quit smoking
☐ The health warnings on cigarette or tobacco packages
☐ Someone from my family or circle of acquaintances got a disease that can be caused by smoking (e.g., cancer)
☐ The fact that smoking is being banned at more and more places
☐ Another main reason

Please continue to the next page!

4.) How long did this most recent quit attempt last before you went back to smoking?
Please choose **only one answer**.

☐ I am still not smoking
☐ Less than a day
☐ Less than a week
☐ Less than a month

5.) Have you consulted your general practitioner since our survey about 4 weeks ago mainly or solely to get behavioural support for smoking cessation? Please choose **only one answer**.

☐ Yes, _____ times (please only state the number of consultations mainly or solely because of your attempt to quit smoking since our last survey)
☐ No

6.) Which of the following products / methods have you tried to assist this last quit attempt?
Multiple answers allowed.

☐ Nicotine replacement product (e.g., nicotine patches or nicotine gums)
☐ The drug Zyban (Bupropion)
☐ The drug Champix (Varenicline)
☐ Behavioural therapy for smoking cessation (e.g., single or group therapy)
☐ Telephone counselling for smoking cessation
☐ E-Cigarettes with nicotine
☐ E-Cigarettes without nicotine
☐ A book on smoking cessation (e.g., "Easyway" by Allen Carr)
☐ An app or internet program for smoking cessation (on a smartphone, tablet or computer)
☐ Acupuncture
☐ Hypnotherapy
☐ Alternative practitioner / naturopath
☐ None of the above mentioned products / methods → **continue with question 9**

Please continue to the next page!

7.) Can you recall how much money you spent <u>in total</u> for these products / methods to assist this <u>last</u> quit attempt? **<u>Multiple answers allowed.</u>**

_______ € for nicotine replacement therapy (e.g., nicotine patches, nicotine gums, nicotine sprays)

_______ € for drugs (e.g., Zyban, Champix)

_______ € for e-cigarettes (if used as an aid to quit smoking)

_______ € for behavioural therapy (e.g., single or group therapy)

_______ € for other treatment (e.g., acupuncture, hypnotherapy, alternative practitioner / naturopath)

_______ € for other products/methods (e.g., book on smoking cessation or chargeable app)

☐ I spent <u>no</u> money → **continue with question 9**

☐ I cannot remember → **continue with question 9**

8.) In case you spent money on one or more of these products / methods during this <u>last</u> quit attempt, did health insurance reimburse any of it?

☐ Yes, a total of _________€

☐ No, I paid everything out of my own pocket

☐ I don't know if my health insurance offers such reimbursement

☐ I did not submit any request for reimbursement to my health insurance

☐ I cannot remember

Please continue to the next page!

The following final set of questions is related to your current
<u>general state of health</u>*

9.) Under each heading, please tick the ONE box that describes your health **TODAY** best. Please choose **<u>only one answer</u>** per heading **<u>A) to E).</u>**

A) MOBILITY / FLEXIBILITY

☐ I have no problems in walking around

☐ I have slight problems in walking around

☐ I have moderate problems in walking around

☐ I have severe problems in walking around

☐ I am unable to walk around

B) SELF-CARE

☐ I have no problems washing or dressing myself

☐ I have slight problems washing or dressing myself

☐ I have moderate problems washing or dressing myself

☐ I have severe problems washing or dressing myself

☐ I am unable to wash or dress myself

C) USUAL ACTIVITIES (e.g., work, study, housework, family or leisure activities)

☐ I have no problems doing my usual activities

☐ I have slight problems doing my usual activities

☐ I have moderate problems doing my usual activities

☐ I have severe problems doing my usual activities

☐ I am unable to do my usual activities

D) PAIN / DISCOMFORT

☐ I have no pain or discomfort

☐ I have slight pain or discomfort

☐ I have moderate pain or discomfort

☐ I have severe pain or discomfort

☐ I have extreme pain or discomfort

Please continue to the next page!

E) ANXIETY / DEPRESSION

☐ I am not anxious or depressed

☐ I am slightly anxious or depressed

☐ I am moderately anxious or depressed

☐ I am very anxious or depressed

☐ I am extremely anxious or depressed

10.) We would like to know how good or poor your health **TODAY**.

- Imagine a scale (like a standing up ruler)
- The top of the scale is marked with 100, the bottom with zero (0)
- 100 (top) means the best health you can imagine.
  0 (bottom) means the worst health you can imagine.
- Mark an X on the scale to indicate how is your health today
- Now, please note the number your marked in the box below.

YOUR HEALTH TODAY = ☐

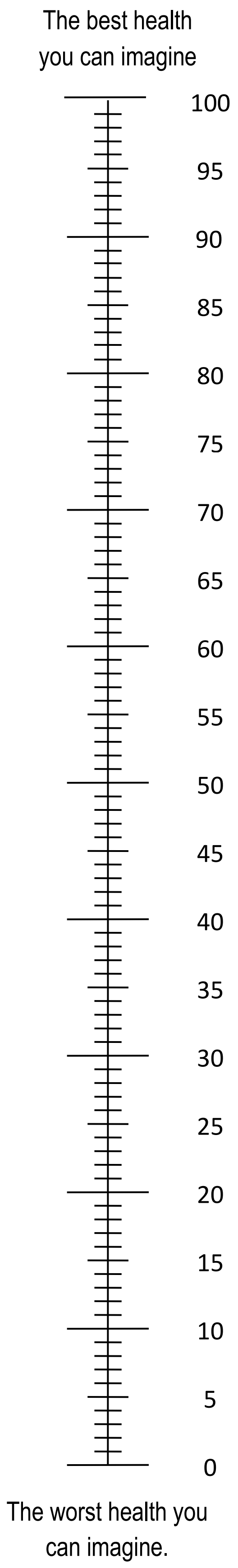

*Rabin R, de Charro F. EQ-5D: a measure of health status from the EuroQol Group. Ann Med 2001;33:337-43.
